# Grain and Domain Microstructure in Long Chain *N*-Alkane and *N*-Alkanol Wax Crystals

**DOI:** 10.1021/acs.cgd.4c00909

**Published:** 2024-12-07

**Authors:** Emily Wynne, Simon D. Connell, Rachael Shinebaum, Helen Blade, Neil George, Andy Brown, Sean M. Collins

**Affiliations:** † School of Chemical and Process Engineering, University of Leeds, Woodhouse Lane, Leeds LS2 9JT, U.K.; ‡ Bragg Centre for Materials Research, University of Leeds, Woodhouse Lane, Leeds LS2 9JT, U.K.; § School of Physics and Astronomy, University of Leeds, Woodhouse Lane, Leeds LS2 9JT, U.K.; ∥ AstraZeneca, Technical Operations Science & Innovation, Pharmaceutical Technology & Development, Operations, Macclesfield SK10 2NA, U.K.; ⊥ AstraZeneca, Oral Product Development, Pharmaceutical Technology & Development, Operations, Macclesfield SK10 2NA, U.K.; # Syngenta, Jealott’s Hill, Warfield, Bracknell RG42 6EY, U.K.; ∇ School of Chemistry, University of Leeds, Woodhouse Lane, Leeds LS2 9JT, U.K.

## Abstract

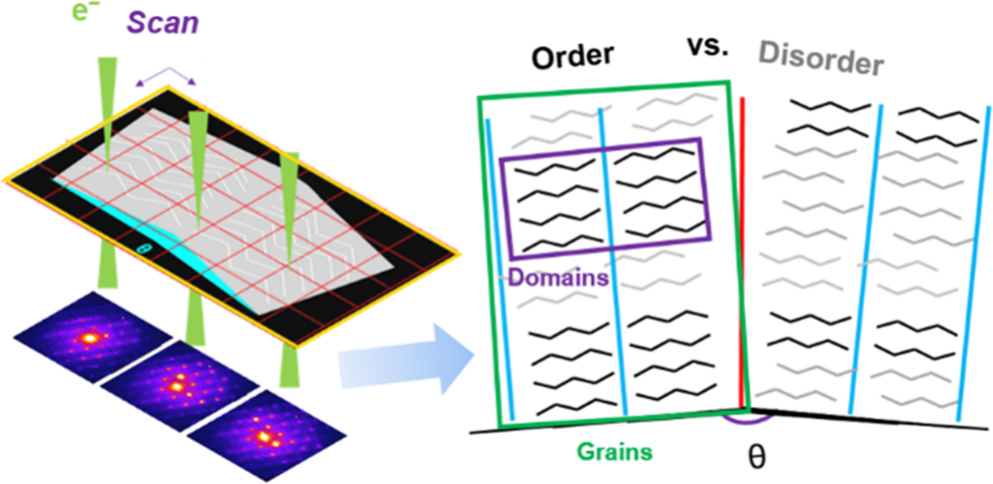

Waxes comprise a diverse set of materials from lubricants and coatings to biological materials such as the intracuticular wax layers on plant leaves that restrict water loss to inhibit dehydration. Despite the often mixed hydrocarbon chain lengths and functional groups within waxes, they show a propensity for ordering into crystalline phases, albeit with a wealth of solid solution behavior and disorder modes that determine chemical transport and mechanical properties. Here, we reveal the microscopic structure and heterogeneity of replica leaf wax models based on the dominant wax types in the *Schefflera elegantissima* plant, namely C_31_H_64_ and C_30_H_61_OH and their binary mixtures. We observe defined grain microstructure in C_31_H_64_ crystals and nanoscale domains of chain-ordered lamellae within these grains. Moreover, nematic phases and dynamical disorder coexist with the domains of ordered lamellae. C_30_H_61_OH exhibits more disordered chain packing with no grain structure or lamellar domains. Binary mixtures from 0–50% C_30_H_61_OH exhibit a loss of grain structure with increasing alcohol content accompanied by increasingly nematic rather than lamellar chain packing, suggesting a partial but limited solid solution behavior. Together, these results unveil the previously unseen microstructural features governing flexibility and permeability in leaf waxes and outline an approach to microstructure analysis across agrochemicals, pharmaceuticals, and food.

## Abstract 2

The nanoscale structure of waxes determines their function in materials from coatings to the leaves of plants but remains poorly documented. This study reveals a hierarchical microstructure (of grains and domains) tuned by alcohol content, outlining the basis of composition-dependent properties.

## Introduction

Waxes encompass a rich variety of technologically as well as biologically important solids. Some of the simplest waxes, paraffin waxes are used in many products from polishes, lubricants, and waterproof coatings to electrical insulators.^1,2^ Among diverse biological wax materials, the intracuticular wax (IW) layer on plant leaves plays a particularly important role in restricting water loss to protect the plant from dehydration and in other molecular diffusion to and from the leaf surface,^3^ a feature of significant interest in crop plants and in terms of fundamental biological processes. Despite waxes comprising in large part simple linear chain hydrocarbon structures, which arrange in side-by-side chain packing in crystalline forms, they support a great deal of structural variety due to diverse ordering and disordering possibilities. Determining the degree of (dis)ordered structure of these organic materials at the fundamental length scale of a few chain repeats (nanometer-scale) is crucial across a number of sectors for understanding properties such as trans film diffusion, density, congealing point, and hardness,^4^ as well as underpinning understanding of how water and agrochemical active ingredients interact with plant crops.^5^

Here, we focus on a reduced set of long chain hydrocarbons found in leaf waxes. Different plants and varieties have very different compositions, but we focus on the *Schefflera elegantissima* plant, a plant with a simple IW layer consisting mainly of long chain alkanes and alcohols. Studying this simple system may lead to the discovery of features that can be linked to plant performance across many different species. In leaves, the IW layer’s structure is thought to have a “brick and mortar” type arrangement consisting of highly crystalline packing of chains in “lamellar” blocks or “bricks” surrounded by amorphous regions,^6^ though the precise size and relative ordering of these lamellae has not been established. This arrangement of disorder at chain ends and in the gaps between lamellae is thought to provide high diffusivity pathways through the wax layer. Examination of waxes at the nanoscale is imperative to evaluate this hypothesis. Simplified, replica leaf waxes provide a first route to revealing the structural complexity native to these long chain hydrocarbons and their mixtures.

Paraffin waxes, i.e., long chain alkanes, can be thought of as packing similarly to polyethylene. In polyethylene, the molecular chains pack in an extended linear conformation, but without lamellar ordering as the polyethylene is assumed to exhibit effectively infinite chain extension. Polyethylene is most commonly described by an orthorhombic subcell but can also exhibit monoclinic or triclinic subcells.^7−9^ Finite linear alkanes of *n* carbon atoms in high purity forms, with minimal chain length dispersion, organize into lamellar blocks with gaps in between aligned chain ends at the boundaries of the block or lamella. These *n*-alkane chains pack with chains fully extended and end to end. Molecules of equal length form well-defined layers within these lamellae where the length of the molecule determines the interlamellar spacing.^10^ Polydisperse *n*-alkane (paraffin) waxes readily form solid solutions, lowering the degree of ordering such that paraffins exhibit polymorphism based on chain length, purity, and temperature.^11^ Nevertheless, solid solutions of paraffins will still form single crystals when chain lengths are within four carbon units of each other.

Analytical techniques such as X-ray diffraction (XRD) and atomic force microscopy (AFM) have been used to characterize long chain waxes. Single crystal and powder XRD have been used in early studies of wax structure, such as in structural studies of fatty acids^12^ and *n*-alkanes.^13^ For the *n*-alkanes, seminal predictions of crystal structures^14^ have been verified through synchrotron powder diffraction.^15^ Powder XRD patterns from a typical bulk assembly of microcrystals can provide somewhat limited information about the lamellar stacking of the chain layers and the preferred subcell packing of the polyethylene repeat. It is known that high molecular weight polyethylene can fold to form thin single crystal lamellae with the molecular chains initially shown to be perpendicular to the flat faces of the crystals.^16^ It has since been observed that chain tilt relative to the lamella normal can affect the sharpness of chain folding (affecting the interlamellar distance) and whether chains form isolated or stacked lamellae, with isolated lamellae commonly exhibiting chain tilt angles close to a thermodynamically favored 34° and stacked lamellae exhibiting smaller tilt angles.^17^ Atomic force microscopy (AFM) has revealed defects in paraffin crystals such as spiral growth formed by screw dislocations.^18^ XRD and AFM, however, do not afford insight into the spatial arrangement of lamellar microstructure accessible using transmission electron microscopy (TEM) techniques.^10^

Dorset and colleagues have used selected area electron diffraction (SAED) to extensively characterize and determine the structure of paraffin waxes at the length scale of a few micrometers.^19−23^ Obtaining single crystal diffraction data by SAED accordingly has a much less stringent requirement on crystal size relative to X-ray approaches. However, TEM samples must be sufficiently thin to be electron transparent. Using established procedures for molecular crystal orientation control,^24,25^ Dorset and colleagues prepared electron transparent paraffin single crystals in two key orientations, with chains parallel and with chains perpendicular to the electron beam. These studies have revealed molecular packing symmetry as well as signatures of interlamellar disorder in a variety of single and multicomponent waxes. Although many other orientations on surfaces are conceivable, these two selected orientations follow the symmetry of *n*-alkane and *n*-alkanol molecules in the solid state: The chains parallel to the electron beam offer a unique view of the side-by-side packing of these chains, and the chains perpendicular to the beam presents one of several orthogonal perspectives to examine the key end-to-end packing of chains. Together, these two orientations provide an important set for initial investigations of the wax microstructure in pure *n*-alkanes, *n*-alkanols, and their binary mixtures.

**Figure 1:**
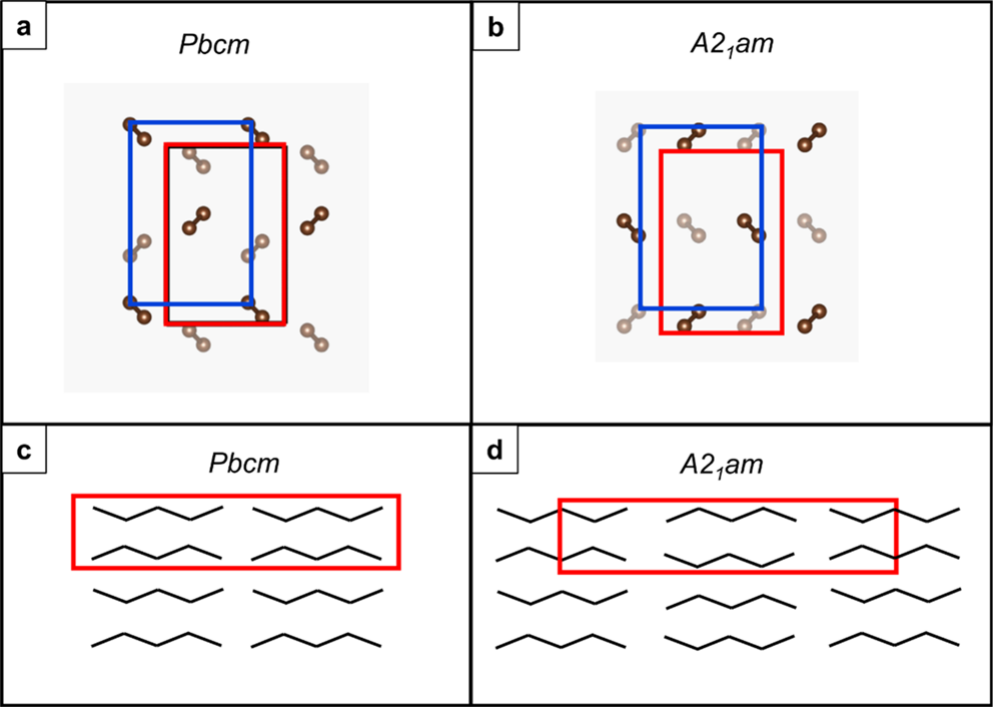
(a, b) Representation of carbon chain packing as seen by the electron beam in the “down-chain” orientation for the alkane packed with (a) *Pbcm* symmetry and (b) *A2*_1_*am* symmetry. (c, d) Representation of carbon chain packing as seen by the electron beam in the “chains-flat” orientation for the alkane packed with (c) *Pbcm* symmetry and (d) *A2*_1_*am* symmetry. The red boxes indicate the unit cell for the indicated space group symmetry, and the blue boxes indicate the polyethylene type cell. Alternate chains are faded to represent the offset of chains in real space in neighboring lamellar layers.

Pure, single-component even and odd *n*-alkanes exhibit distinct packing symmetries, with even *n*-alkanes favoring monoclinic unit cells (and exhibiting the monoclinic or triclinic polyethylene subcell)^10^ and odd *n*-alkanes packing in orthorhombic symmetry (following the orthorhombic polyethylene subcell).^14^ With small amounts of impurities, however, even *n*-alkanes adopt orthorhombic packing which may explain the preponderance of orthorhombic forms with polyethylene-like domains in plant waxes.^10^ Odd *n*-alkanes with 11 or more carbons (*n* ≥ 11) consistently adopt orthorhombic symmetry. Smith reported a structure solution for the *n =* 23 alkane (*n* carbon atoms) with *Pbcm* space group symmetry. In Smith’s structure, the long chain end-to-end packing is along the *c*-axis such that mirror planes at *c*/4 and 3(*c*/4) coincide with the molecular mirror symmetry.^13^ Dorset, in contrast, has reported odd *n*-alkanes packing with *A2*_1_*am* symmetry when prepared with a benzoic acid epitaxial template to yield crystals with chains perpendicular to the electron beam in SAED.^26^ In the *A2*_1_*am* unit cell, the long chains are likewise along the *c*-axis, but with the molecular mirror planes at *c*/*2* and *c*. The difference in chain packing is perhaps most noticeable in two visualizations, depicted in Figure 1: (1) When viewed along chains, i.e., along [001] and here denoted the “down-chain” orientation, the offset between sequentially stacked chains differs (see also Figure S1), and (2) when viewed with the chains flat, i.e., perpendicular to [001] in these orthorhombic unit cells and here denoted the “chains-flat” orientation, the sequential chains along the *c*-axis in the *A2*_1_*am* structure are rotated by 180°.

Irrespective of the precise unit cell for alkane lamellae, additional disordered states have been identified with chains slipping across lamellar regions giving rise to “nematic” phases.^27^ Earlier XRD analysis by Lüth^28^ provides further evidence of this general characteristic, where longitudinal molecular disorder within individual lamellae was proposed to compensate copacking of dissimilar chain lengths.^29^ These “nematic” phases draw parallels with the “brick and mortar” model proposed for the structure of the IW layer comprised of both ordered and disordered regions.

**Figure 2:**
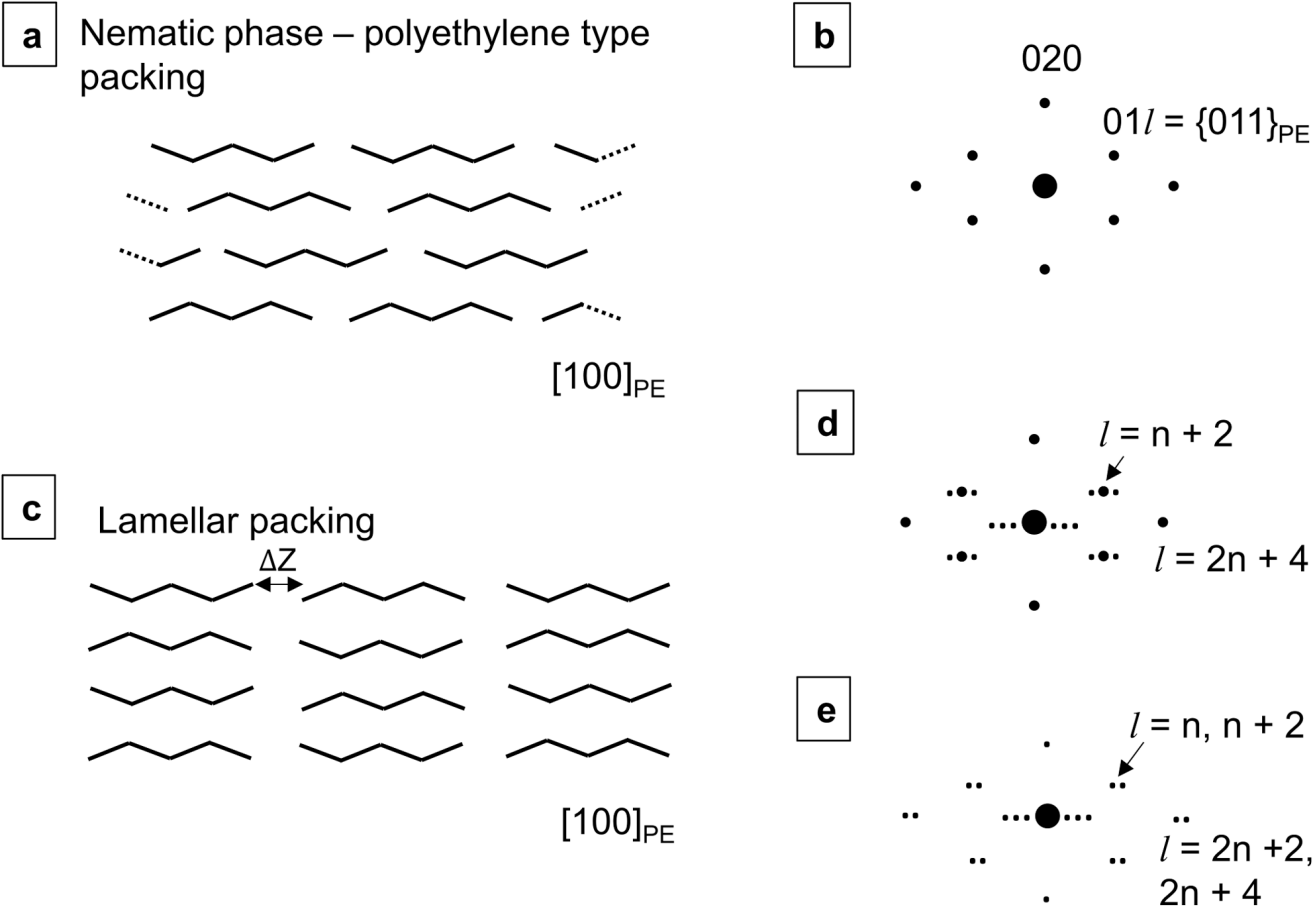
Representation of the *n*-alkane nematic phase shown with carbon chain packing in the “chains-flat” orientation. (b) A schematic representation of the corresponding electron diffraction pattern exhibits a polyethylene-like arrangement of spots with no splitting of the  peaks. (c) Representation of *n*-alkane packing with lamellar ordering shown with carbon chains packing in the “chains-flat” orientation. (d), (e) Schematic representations of the electron diffraction patterns for two cases: (d) If the interlayer spacing is an integer multiple of the half zigzag repeat in the carbon chain, with the distance between lamellae defined as Δ*Z* = 3(*c*_PE_/2) for polyethylene subcell c-parameter *c*_PE_, then the diffraction pattern will contain lamellar reflections with a strong single maxima. (e) If Δ*Z* ≠ 3(*c*_PE_/2), then the  polyethylene reflections split into two reflections.^27^

Electron diffraction is highly sensitive to this chain ordering, particularly for crystals oriented with chains perpendicular to the electron beam, or equivalently with “chains flat” on the support film of a TEM grid. In this latter orientation, if the chains pack with nematic ordering, then the  diffraction pattern strongly resembles that of the orthorhombic polyethylene subcell, with the {011}_PE_ reflections consisting of a single, dominant spot (Figure 2a,b). Here, the subscript PE denotes the polyethylene subcell, and  coincides with  for *n* carbons.^1,27^ When the chains pack with defined lamellae, then the diffraction pattern will contain additional reflections arising from the interlamellar spacing around the strong polyethylene type reflections (Figure 2c–e), with the {} for  particularly pronounced. That is, differences in these additional reflections arise from the symmetry of the space group in unit cell models of each packing arrangement. However, the size and shape of regions with lamellar ordering and their position relative to nematic phases within wax crystals remain obscured by diffraction analysis without coordinated real-space imaging.

To extend electron imaging and diffraction toward understanding wax films on plant leaves, the pure forms and binary mixtures of *n*-alkanes and their simple mixtures provide a critical starting point. The *S. elegantissima* plant’s IW layer contains only four major long chain hydrocarbon species, mainly long chain alkanes and alcohols.^30,31^ Basson and Reynhardt have shown that these long chain alkanes and alcohols, such as *n*-hentriacontane C_31_H_64_ and 1-triacontanol C_30_H_61_OH used in this study, can be used to form replica leaf wax systems.^32^ As such, we focus on these two components and their binary mixtures.

**Figure 3:**
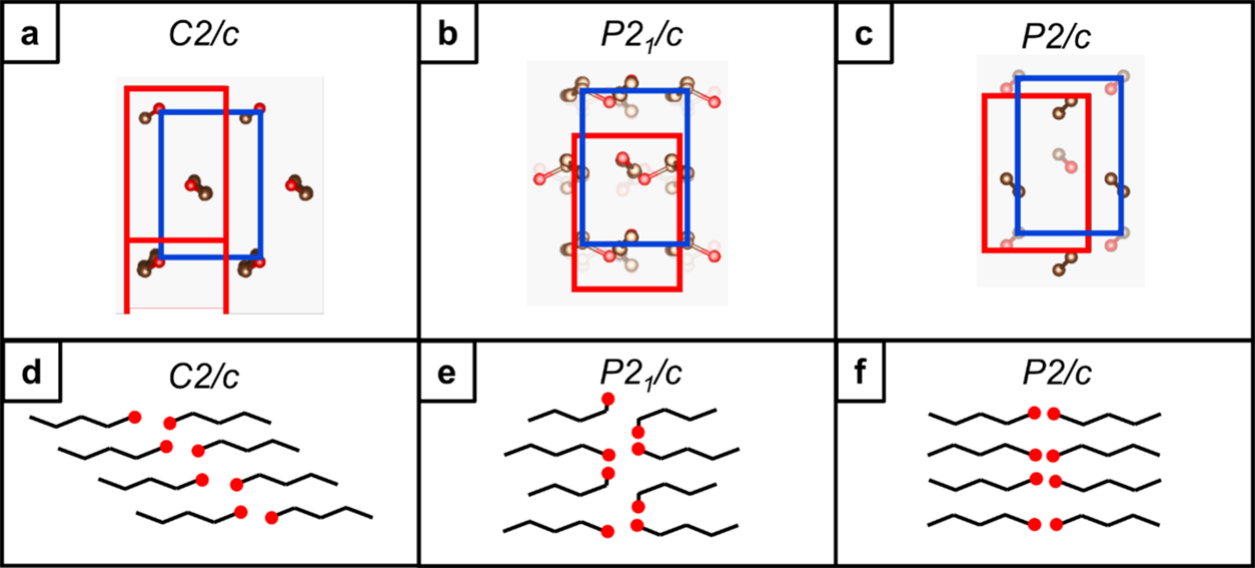
(a–c) Representation of carbon chain packing as seen by the electron beam in the “down-chain” orientation for the alcohol packed with (a) *C2*/*c* symmetry, (b) *P2*_1_/*c* symmetry and (c) *P2*/*c* symmetry. (d–f) Representation of carbon chain packing as seen by the electron beam in the “chains-flat” orientation for the alkane packed with (d) *C2*/*c* symmetry, (e) *P2*_1_/*c* symmetry and (f) *P2*/*c* symmetry. The red boxes indicate the unit cell, and the blue boxes indicate the polyethylene type cell.

Long chain primary alcohols (*n*-alkanols) are known to pack differently to the *n*-alkanes. A key distinction arises from the asymmetry of the chain end containing the alcohol moiety. The alcohol groups from two chains form hydrogen bonding interactions, driving alcohol–alcohol interactions as a guiding organizational principle for *n*-alkanol crystallization. Typical packing motifs are described by either a monoclinic γ form (Figure 3a, *C2*/*c* unit cell), a “staircase” packing with chain ends offset rather than aligned,^33^ or by a monoclinic β form with the chain ends aligned in lamellae but with *gauche* conformations at the chain ends to accommodate a hydrogen bonding network between the terminal alcohol groups (Figure 3b, *P2*_1_/*c* unit cell).^34^ Dorset has proposed long chain alcohols may also pack in an orthorhombic β form, with similar structural characteristics to the *n*-alkanes.^35^ We have constructed a *P2*/*c* unit cell following the *Pbcm* structure of the *n*-alkanes to model these characteristics (Figure 3c). In the “down-chain” orientation, all considered structures exhibit packing as in the polyethylene subcell, albeit with a variety of displacements akin to differences between the *Pbcm* and *A2*_1_*am n*-alkane unit cells. These structural models, built from previous reports on shorter *n*-alkanols, provide an overview of the key functional group interactions and resulting packing motifs possible in C_30_H_61_OH and in binary mixtures with C_31_H_64_.

A major obstacle to the TEM study of these wax materials is the high sensitivity of organic materials to the electron beam and their high susceptibility to damage, in particular by radiolysis.^10,36−38^ Long chain alkanes have previously been reported to undergo significant damage at a cumulative fluence as low as 5–10 e^–^/Å^2^ under 300 keV irradiation,^39^ but recent advances in low-dose electron microscopy techniques have enabled improved access to nanoscale resolution in organic molecules.^40,41^ Low-dose is typically defined as <10 e^–^/ Å^2^ for biological and organic materials.^42,43^ Scanning electron diffraction (SED) has particularly demonstrated suitability for low-dose operation. In SED, a nearly parallel, low convergence angle probe is scanned across a sample at nanometer-scale steps while two-dimensional (2D) diffraction patterns are simultaneously acquired at every probe position. A simplified schematic of this four-dimensional scanning transmission electron microscopy (4D-STEM) technique is presented in Figure S2. By combining imaging and diffraction data at nanometer spatial resolution (<5 nm) using low beam currents (∼1 pA) and fast detector readout (∼1 ms), SED has advanced real and reciprocal space low-dose structural analyses of beam sensitive materials, including orientation mapping of semicrystalline polymers,^17,44^ defect mapping in metal–organic frameworks (MOFs),^45^ and resolving the local crystalline nanostructure in organic molecular crystals.^46,47^

In this study we use a beam energy of 300 keV, a beam current of 1 pA, a dwell time of 1 ms, and a beam convergence angle of <1 mrad at room temperature to minimize radiation damage from radiolysis. These conditions offer a trade-off between the reduction of beam damage (given lower rates of radiolytic damage are observed in organic materials at higher beam energies)^48^ while still achieving moderate diffraction resolution to distinguish the Bragg reflections (diffraction disk radius α/λ ∼ 0.05 Å^–1^ for convergence semiangle α and de Broglie wavelength λ) while also retaining a nanometer-scale probe. These conditions give a probe fluence of ∼5 e^–^/Å^2^. We assess the effect of the accompanying dose (energy transfer) to the selected *n*-alkane and *n*-alkanol materials through cumulative fluence measurements to identify the specific critical fluence for loss of the crystalline structure under electron beam exposure. We note that while cooling (e.g., to 100 K with liquid nitrogen cooling) offers an observed reduction in beam damage, such improvements are of the order of a factor of 2^48^ while also introducing additional practical complexities. For SED measurements where the fluence can be controlled to minimize structural changes under the beam during an experiment, room temperature conditions also avoid any ambiguity in temperature-dependent phase transitions. Here, we detect Bragg spots out to scattering angles corresponding to *d*-spacings of 1.19 and 0.87 Å, in the “down-chain” and “chains-flat” orientations, respectively, confirming we retain high-resolution structural information under these conditions.

Here we present a combined TEM, AFM, and SED approach for the microscopic analysis of C_31_H_64_ and C_30_H_61_OH crystals and their binary mixtures. We first identify the characteristics of the pure C_31_H_64_ and C_30_H_61_OH crystal structures by TEM and AFM. Then, through SED we identify the nanoscale spatial arrangement of chain-ordered lamellae within the pure components and evaluate lamellar structure, lamellar size, and interlamellar disorder. These results outline microstructural models consisting of hierarchical grain structure containing domains with highly ordered lamellar packing. In turn, we discuss the trends in lamellar packing and ordering across a series of binary mixtures. Together, these findings describe the native structural heterogeneity in replica leaf waxes at the nanoscale, providing new insights for models of water vapor and other molecular diffusion through IW layers as well as microstructural principles for the examination of wider wax structure–function relationships.

## Materials and Methods

### Sample Preparation

Samples of *n*-hentriacontane (C_31_H_64_) were purchased from Sigma-Aldrich with >98% reported purity. Samples of 1-triacontanol (C_30_H_61_OH) were purchased from Caymen Chemicals with >98% reported purity. Binary mixtures were prepared using a molar fraction of the two components melted together and allowed to cool and crystallize to homogenize the samples prior to any subsequent melting.

Samples were prepared following methods outlined by Fryer^11^ and Wittmann^24^ on continuous amorphous carbon supported on a copper mesh grids. The first method of preparation was drop-casting from solution. One mg of the long chain hydrocarbon was partially dissolved in 30 μL hexane to form a supersaturated solution. 2 μL was dropped on to a continuous carbon film supported on a copper 300 mesh grid and the hexane left to evaporate naturally, leaving crystalline regions of the product on the grid, giving chains orientated in the “down-chain” orientation.

The second method also outlined in Fryer^11^ and Wittmann^24^ relies on mixing the paraffin in the solid state with a large excess of benzoic acid crystals, melting the mixture and crystallizing the paraffin on the benzoic acid crystals to template the growth of extended plate-like wax crystals with the long hydrocarbon chain in the plane of the plates. Briefly the paraffin-benzoic acid mixture was enclosed between two glass slides. A flat aluminum bar with one end on a hot plate, (hot plate heated to 100 °C) provided the temperature gradient, and the glass “sandwich” was slid slowly (∼1 cm per second) up and down the bar causing the mixture to melt and then solidify. The mixture was determined to have melted when the mixture changed from a white solid powder mixture to a colorless liquid mixture between the two glass slides. Recrystallization was identified by the reformation of white crystals within a few seconds of cooling. Once cool, the two sheets were separated, and the two layers of the mixture obtained were floated on to a water surface. The benzoic acid dissolved in the water and the remaining wax specimens were picked up on TEM grids. This procedure resulted in paraffin crystals supported on the carbon film of the TEM grid, giving chains lying flat on the TEM grid support film surface. While both methods (drop-casting and templated methods) produce crystals with some variation in thickness, the methods consistently produce electron transparent crystals exhibiting similar diffraction characteristics within a given sample (composition and preparation method).

### Unit Cell Structural Model Building

The *Pbcm* and *A2*_1_*am* C_31_H_64_, and *P2*_1_/*c* and *P2*/*c* C_30_H_61_OH unit cell models were built by extending the repeating pattern of carbon atomic coordinates in reported structures for shorter chain hydrocarbons. The *Pbcm* C_31_H_64_ structure was adapted from the structure for tricosane (C_23_H_48_) reported by Smith.^13^ The *A2*_1_*am* C_31_H_64_ was in turn adapted from the carbon spacings in the *Pbcm* cell by shifting the atomic coordinates to the symmetric positions outlined for alternative setting *Bb2*_1_*m* by Lüth et al.^28^ followed by necessary axis adjustments. Lattice parameters were defined for the *Pbcm* and *A2*_1_*am* unit cells using experimental data. The *C2*/*c* C_30_H_61_OH structure was adapted from the structure for *n*-eicosanol (C_20_H_41_OH) reported by Michaud et al.^49^ The *P2*_1_/*c* C_30_H_61_OH structure was adapted from the structure for 1-heptadecanol (C_17_H_35_OH) reported by Seto.^34^ The *P2*/*c* C_30_H_61_OH structure was adapted from the *Pbcm* unit cell by reducing the space group symmetry and asymmetric substitution of the terminal C for O. The lattice parameters for the *P2*_1_/*c* and *C2*/*c* unit cells were calculated by extending the unit cells so that the previous C–C and O–O distances (in Å) were retained.

In order to carry out electron diffraction simulations to illustrate the effects of dynamical disorder, we built a simple model of unidirectional chain sliding for kinematical diffraction calculations. Toward this end, supercell structures of the C_31_H_64_*A2*_1_*am* were constructed to support fine-scale sampling of scattering vectors  in reciprocal space. First, the space group symmetry was reduced to *P*1. This unit cell was then extended along the *b*-axis by 10, 15, and 20 repeat unit cells using Vesta software. Chains were displaced along the *c*-axis by shifting the atomic coordinates of one molecule in the supercell by a set value smaller than the C–C bond distance (0.83 Å displacement along the *c*-axis, compared to 1.28 Å between adjacent carbon atoms along the *c*-axis). These structures represent the characteristic type of symmetry breaking in snapshots of molecular sliding motion. More complete simulations are possible with distributions of displacements and using dynamical scattering calculations.^50^ However, this simple model provides the necessary elements for reproducing the qualitative characteristics of the diffuse scattering arising from disorder in the form of unidirectional displacement.

For zone axis pattern simulations (Figures S5, S6, and S8) electron diffraction patterns were simulated using SingleCrystal, part of the CrystalMaker software. For patterns away from high symmetry zone axis orientations (Figure S18), electron diffraction patterns were simulated using the DiffSims package as part of Pyxem to incorporate effects of the Ewald sphere. Briefly, in Pyxem (version 0.11.0), diffraction is modeled by the intersection of the Ewald sphere (set by the beam energy) and a reciprocal lattice given by a unit cell (including the atom basis), where the finite extent of the reciprocal lattice sites are modeled by an allowed excitation error (accounting for the shape factor for thin, electron-transparent crystals). Intensities are modeled at each reciprocal lattice site by the kinematical intensity (square of the modulus of the structure factor) and modified according to the excitation error relative to the Ewald sphere for a given orientation of the sample relative to the electron beam. Crystal orientations in simulations were defined by the unit cell direction [UVW] along the electron beam trajectory.

### Microscope Apparatus

TEM characterization was performed at room temperature by using an FEI Titan^3^ Themis operated at a 300 kV accelerating voltage. The FEI Titan^3^ Themis S/TEM microscope was equipped with a high-brightness “X-FEG” electron gun, an S-TWIN objective lens, a Gatan OneView 4K CMOS digital camera and a continuously variable gun lens to control the beam current. “Low-dose” TEM was achieved by spreading the monochromator lens to reduce the electron flux to less than 1 e^–^/Å^2^ s.

SED data were acquired using a JEOL ARM300CF fitted with an ultrahigh-resolution pole piece, a cold field emission gun, and aberration correctors in both the probe forming and image-forming optics (Diamond Light Source, U.K.). The instrument was operated at 300 kV. A nanobeam configuration was obtained by switching off the aberration corrector in the probe forming optics and using a 10 μm condenser aperture to obtain a convergence semiangle <1 mrad and a diffraction-limited probe diameter of approximately 5 nm. The probe current was measured using a Faraday cup as 1 pA, and the exposure time was 600 μs or 1 ms per probe position. The estimated electron fluence for 600 μs dwell time, assuming a disk-like probe, was ∼5 e^–^/Å^2^. A diffraction pattern was acquired at every probe position using a Merlin-Medipix hybrid counting-type direct electron detector (Quantum Detectors, U.K.).

Data processing was carried out using the Pyxem open-source Python library for multidimensional diffraction microscopy.^51^ Calibration data were recorded from standard MoO_3_ and Au-cross-grating samples as reported previously.^52^ Briefly, the rotation between the scan direction and the diffraction pattern was calibrated by identifying the angle between the characteristic long axis of MoO_3_ crystals of known habit and the corresponding diffraction spots. An Au-cross-grating with a 500 nm period was used to calibrate the image pixel size. By recording an SED data set from polycrystalline Au, a diffraction pattern consisting of characteristic rings was acquired and fitted to a set of ellipses to determine residual elliptical distortions and to determine the diffraction camera calibration. For long chain hydrocarbon samples, the direct beam was first aligned to pixel precision and then centered using a cross-correlation function in Pyxem to subpixel precision. An affine transformation matrix was used to remove the measured elliptical distortions and the pattern rotation relative to the scan direction.

Diffraction patterns, originally 515 × 515 pixels, were cropped to 514 × 514 pixels and then binned by a factor of 2 to produce diffraction patterns with dimensions 257 × 257 pixels. Low-angle annular dark field (ADF) images were formed by integrating the diffraction pattern at each probe position between an inner radius of 2θ = 2.1 mrad and an outer radius of 2θ = 7.5 mrad to produce an image dominated by diffraction contrast. Medium-angle ADF images, denoted ADF-1 images, were formed by integrating the diffraction pattern at each probe position between an inner radius of 2θ = 12.0 mrad and an outer radius of 2θ = 26.0 mrad, (beyond the scattering angles observed for the first order Bragg diffraction spots) to form an image dominated by mass–thickness contrast. A second set of medium-angle ADF images, denoted ADF-2 images, were formed by integrating the diffraction pattern at each probe position between an inner radius of 2θ = 16.2 mrad and an outer radius of 2θ = 26.0 mrad, similarly to form an image with greater contributions from mass–thickness contrast. Average electron diffraction patterns were obtained by taking the mean intensity from all diffraction patterns contributing to an image. Virtual dark field (VDF) images were produced in Pyxem by defining regions of interest in the diffraction pattern and integrating the signal from these regions. Simulated diffraction patterns were produced using the DiffSims package as part of Pyxem by defining a crystal orientation by its direction [UVW] along the electron beam trajectory. Diffraction patterns are presented as the square root of recorded intensity (applied in ImageJ software) for improved visualization of high and low intensity features simultaneously. Poisson distribution statistics for signal-to-background evaluation were carried out using the Scipy Python package. Grain size measurement was carried out in ImageJ.

### AFM Data Collection

Atomic Force Microscopy (AFM) images were acquired using a Dimension FastScan-Bio (Bruker) on a Nanoscope V controller. Peak Force tapping in air was performed using ScanAsyst Fluid probes (Bruker) with a spring constant of approximately 0.60 N/m (probes were calibrated individually using the thermal noise method), a peak force amplitude of 150 nN, a set point of 1.2 nN, a peak force tapping frequency of 1 kHz and a scan rate of approximately 0.5–2 Hz, depending upon image size and resolution. Deflection sensitivity was calibrated on a sapphire sample prior to measuring the sample. Data were acquired in Quantitative Nanomechanical Mode, including the adhesion channel. Although tip radius was not calibrated (a nominal value of 5 nm was input) this does not affect any measurements presented. All data were processed and analyzed using Nanoscope Analysis 3.0. In order to preserve an accurate representation of the three-dimensional (3D) terrace structure and subsequent line sections, the images were initially leveled with a 0-order line fit (i.e., a DC offset) to eliminate z-drift between scan lines, and then flattened with a one-dimensional (1D) plane-fit using a manually defined reference plane encompassing a single flat terrace region, made possible due to the inherent linearity of the FastScan scanner.

### Critical Fluence Determination

Critical fluence values were assessed by recording a time-series of SAED patterns at constant electron flux (fixed beam current) to track the decay of Bragg spot intensity with cumulative electron fluence. SAED Bragg diffraction spot decay measurements were taken on the pure phase alkane and pure phase alcohol samples and a 50:50 binary mixture of the two components in the “down-chain” orientation. Based on critical fluence values for paraffins with similar chain lengths reported in the literature, the electron beam flux was set up to support recording 20–30 data points containing observable diffraction intensities. All diffraction pattern series were acquired using the Titan microscope. An electron beam flux of 0.03 e^–^/Å^2^ s at a fixed magnification was used giving a screen current of 0.076 nA. These values were calibrated using a Faraday cup. The cumulative electron fluence can be calculated by multiplying the time the sample has been exposed to the electron beam (in s) by the electron flux (*J*):
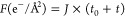
where *J* is the electron flux (e^–^/Å^2^ s), *t*_0_ is the time representing the initial electron exposures including the time taken to record an initial image and an initial diffraction pattern (s), and *t* is the subsequent acquisition time of the following time-series diffraction patterns (s).

The critical fluence can be determined by assuming an exponential decay of diffraction intensity with accumulated fluence:
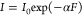
where *I* is the recorded intensity in a given time-series frame, *I*_0_ is the initial intensity in the first frame, *F* is the cumulative fluence, and α is the decay rate constant. Taking the natural logarithm of the maximum intensity of the spots in each pattern linearizes the exponential relationship. The positive reciprocal of the gradient is then taken as the critical fluence.

## Results and Discussion

### Orientation Control in Thin Wax Crystals

**Figure 4:**
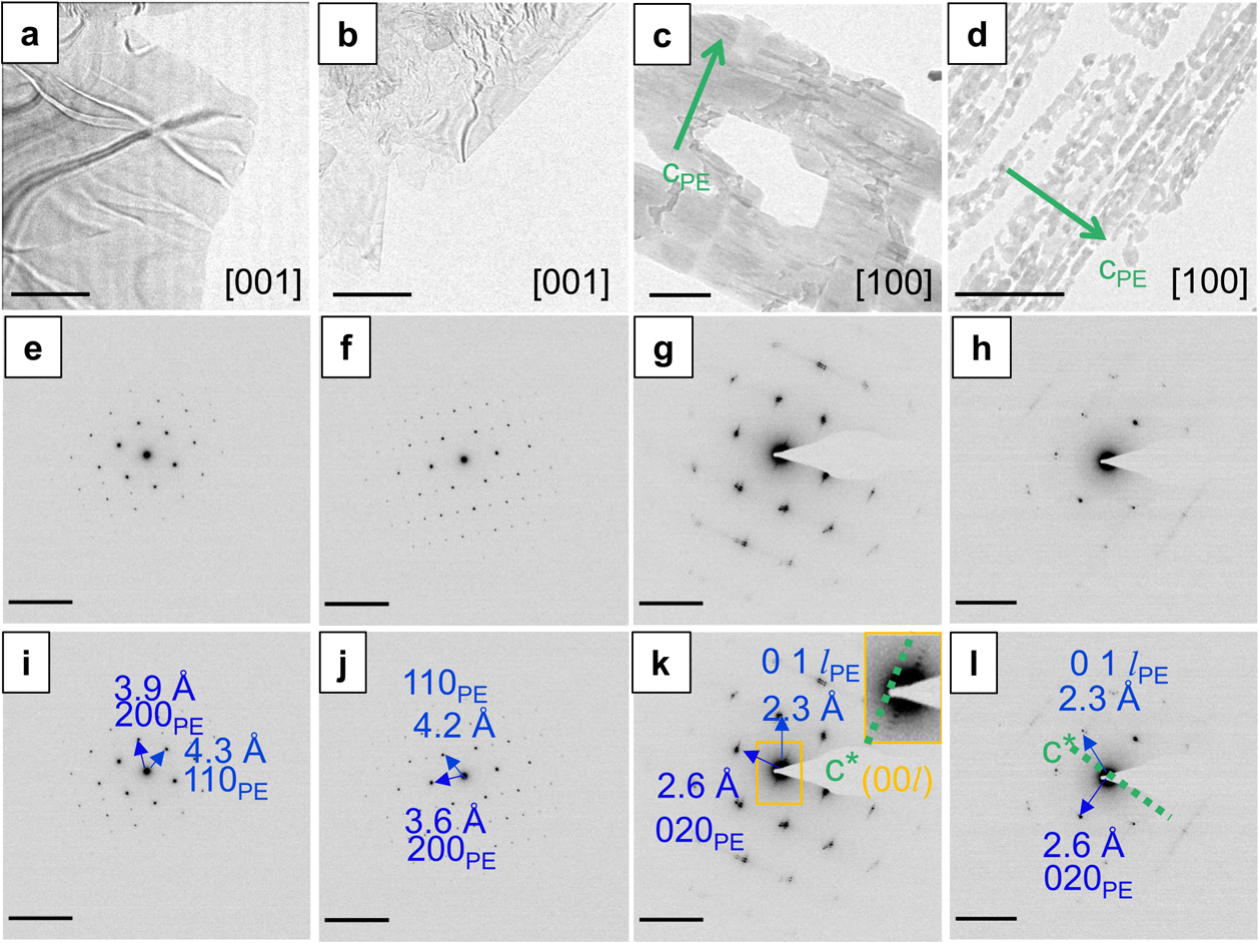
(a–d) Bright field (BF) TEM images and (e–l) SAED patterns, indexed in (i–l), for (a, e, i) C_31_H_64_ prepared to align the alkane chains parallel to the electron beam (“down-chain” orientation), (b, f, j) C_30_H_61_OH prepared in the “down-chain” orientation, (c, g, k) C_31_H_64_ prepared to align alkane chains perpendicular to the electron beam (“chains-flat” orientation), and (d, h, l) C_30_H_61_OH prepared in the “chains-flat” orientation. Green arrows indicate the *c*-axis direction (alkane chain axis) following the polyethylene cell (*c*_PE_). Arrows overlaid on the SAED patterns mark the indexation of the patterns to the PE cell in the [001]_PE_ “down-chain” orientation and the [100]_PE_ “chains-flat” orientation. The inset in (k) shows an expanded section of the SAED pattern marked by the yellow box in (k), with the c*_PE_ reciprocal lattice direction of the *c*-axis marked by the green dashed line. BF image scale bars indicate 2 μm. SAED pattern scale bars indicate 0.5 Å^–1^.

Figure 4 presents conventional TEM analysis of single-component C_31_H_64_ and C_30_H_61_OH crystals. The crystals were prepared following established procedures^24^ to produce crystals either with chains perpendicular to the support film (parallel to the electron beam), denoted the “down-chain” orientation, or with chains parallel to the support film (perpendicular to the electron beam), denoted the “chains-flat” orientation. Measurements were taken under “low-dose” conditions to minimize beam damage to the samples, due to similar long chain alkanes undergoing significant damage at low electron fluence.^39^ Bright field (BF) TEM images show contrast arising primarily from diffraction, with darker regions reflecting greater diffraction. Crystals formed in a “down-chain” orientation exhibited well-defined facets with commonly observed facet angles of ∼109° (Figure 4a,b). Additional dark and light ripples across the crystals were attributed to bend contours, specific regions of the crystal where bending on the support film modifies the diffraction condition. While we have not quantified the thickness of the samples, the crystals are consistently electron transparent, exhibit diffraction to several diffraction orders, and show only minor evidence of dynamical scattering effects in diffraction (e.g., kinematically forbidden diffraction intensities). Our ultimate purpose in this work is to reveal grain and domain structure within crystalline plates, observations not substantially affected by thickness variations between plates.

The corresponding SAED patterns showed a set of four equidistant spots within a consistent set of six strong lowest-angle reflections (Figure 4e,f), showing two perpendicular mirror planes marking out two principal symmetry axes. These first order reflections can be indexed with respect to the polyethylene cell as (110)_PE_, with a *d*-spacing distance of ∼4.3 Å, and (200)_PE_, with a *d*-spacing distance of ∼3.9 Å, where PE denotes the polyethylene cell. We use the PE notation here as a common set of reference points despite differences arising from space group symmetry; the space group symmetry will modify the set of reflections observed (Figures 1–3 and discussion below). The patterns were therefore indexed to the [001]_PE_ zone axis, i.e., with the electron beam direction parallel to [001]_PE_. The corresponding crystal facets were indexed to {110}_PE_ planes, suggesting a facet angle of approximately 113°, broadly consistent with the 109° angle measured from the bright field TEM image. Figures S3–S4 depict the corresponding unit cell orientations in the considered space group symmetries for *n*-alkanes (*Pbcm* and *A2*_1_*am)* and *n*-alkanols (*C2*/*c, P2*_1_/*c*, and *P2*/*c)*.

Crystals formed in a “chains-flat” orientation were prepared via a comelt templating method to direct the crystallization of the hydrocarbons epitaxially on excess benzoic acid. These crystals appear as thin films with a rectangular or block type shape (Figure 4c,d). SAED patterns from these crystals (Figure 4g,h) showed strong reflections at *d*-spacings of 2.6 Å, assigned to the (020)_PE_ spacing, and 2.3 Å, assigned to (011)_PE_ or equivalently () reflections with  for *n* atoms (C + O) in the chain. The patterns were therefore indexed to the [100]_PE_ electron beam direction. Figures S3–S4 highlights the visibility of end-to-end alkyl chain packing in the considered space group symmetries for *n*-alkanes (*Pbcm* and *A2*_1_*am)* and *n*-alkanols (*C2*/*c, P2*_1_/*c, and P2*/*c*). The long axis of the chains (*c*-axis in the PE and all *n*-alkane unit cells) coincides with the short length of the approximately rectangular film blocks (green arrows, Figure 4c,d).

We have also sought to index SAED patterns to the specific unit cells for C_31_H_64_ and C_30_H_61_OH where spots (reflections) additional to those observed in the PE subcell arise, providing insight into the specific chain packing. For C_31_H_64_, there remains some ambiguity in the preceding literature on whether the alkane chains adopt *Pbcm* or *A2*_1_*am* space group symmetry. Figure S5 explores the differences in reflections observed in the corresponding diffraction patterns when considering the different space group packing. If the alkane chain packs with *A2*_1_*am* symmetry, the (110) reflections should be symmetry forbidden when viewed along [001]. The appearance of these reflections in our data may suggest the “down-chain” samples exhibit *Pbcm* space group symmetry rather than the *A2*_1_*am* packing reported by Dorset (Figure 1). Notably, both orientations closely resemble SAED patterns reported by Dorset and colleagues. However, the (110) reflections are symmetry allowed in *Pbcm* whereas very similar spots can be seen in simulated SAED patterns for the *A2*_1_*am* structure when viewing down the [011] axis. This axis is only a small 3° tilt from the [001] axis (Figure S5). The patterns simulated from the *Pbcm* [001] direction and the *A2*_1_*am* structure [011] direction are not readily distinguishable. The appearance of strong bend contours (Figure 4a,b) indicates there is some variation in the crystal orientation across the field of view, and so contributions from *A2*_1_*am* {111} reflections cannot be excluded. In either case, these crystals are in the “down-chain” orientation.

A similar ambiguity in the literature surrounds long chain alcohol packing. Following the solved structure for γ-form *n*-eicosanol,^49,53^ we have constructed a monoclinic γ-form unit cell for C_30_H_61_OH (see also Figure 3). The unit cell has lattice parameters *a* = 132.9 Å, *b* = 4.9 Å, *c* = 9.0 Å, and β = 93.0° and exhibits staggered chain ends or “staircase” packing. The C_30_H_61_OH SAED pattern can be indexed to the [1̅ 0 9] direction in this crystal packing (Figures S6, S7). However, if the chains were to pack with this staircase symmetry, a crystal sitting flat on a solid support (as used here in TEM and AFM) would not appear in the “down-chain” orientation as the chains would be tilted relative to a planar support. The experimental observation of such a “down-chain” orientation suggests the chains pack with more defined lamellar, end-to-end packing, as in the alkane. This arrangement would resemble the known monoclinic β-form (*P2*_1_*/c* space group symmetry), such as that reported by Seto for C_17_H_35_OH,^34^ or an orthorhombic β-form, as suggested by Dorset.^35^ Accordingly, we have built C_30_H_61_OH β-form unit cells (see also Figure 3). The C_30_H_61_OH SAED pattern can be indexed to the [001] direction in both of these structures (Figure S5). The intensities of the (110)_PE_ type reflections in the *P2*_1_/*c* simulated SAED pattern have a lower intensity than the (200)_PE_ reflections (Figure S6), which matches the measured experimental intensities for these reflections in Figure 4f. The *P2*_1_/*c* unit cell, therefore, appears to offer the best unit cell description of the “down-chain” C_30_H_61_OH crystals.

Turning to the “chains-flat” orientation, SAED patterns from C_31_H_64_ crystals were indexed to the *A2*_1_*am* unit cell unambiguously. Figure S4 shows simulated patterns using the *Pbcm* and *A2*_1_*am* structures viewed along [010]_*Pbcm*_ and [100]_*A2*1*am*_, respectively. At first glance, these patterns appear very similar in terms of symmetries and interplanar spacings (*d*-spacings). Inspection of simulated patterns overlaid on top of the experimental SAED patterns from C_31_H_64_ crystals, however, reveals marked differences. In the *Pbcm* pattern, the  reflection appears as a single strong peak at . In the *A2*_1_*am* pattern, the  reflection splits into two strong peaks at  and , in agreement with previous reports by Dorset and colleagues of strong reflections at  and  for *n* carbons in the alkyl chain.^30^ Whereas the simulated SAED pattern for the *A2*_1_*am* unit cell matches the experimental pattern, the simulated SAED pattern for the *Pbcm* unit cell shows a spot midway between the two experimental intensity maxima (Figure S8). The crystals prepared in the “chains-flat” orientation are therefore assigned to the *A2*_1_*am* space group and the patterns were indexed to the [100]_*A2*1*am*_ zone axis (Figure S7). While we do not exclude the possibility that benzoic acid templating induces a different molecular packing arrangement to the packing arising from solution crystallization, we focus here on the observation of lamellar ordering only visible in the “chains-flat” orientation, and we draw attention to the changes induced by the incorporation of C_30_H_61_OH in like-for-like samples.

Figure 4g shows  spots for C_31_H_64_ crystals, further confirming the “chains-flat” crystals exhibit lamellar layers with well-ordered end-to-end packing. Dorset and colleagues have noted the relative intensity of these spots as a marker of deviations from well-ordered lamellae in favor of nematic phases.^27^ These spots are notably mostly absent in the SAED patterns of C_30_H_61_OH in the “chains-flat” orientation (Figure 4h).

Based on the C_30_H_61_OH *C2*/*c* unit cell with “staircase” packing, the  are expected to be absent. The experimental data shows good agreement with a simulated diffraction pattern from the C_30_H_61_OH *C2*/*c* unit cell oriented along [2 0 45], confirming the chains lie flat on the support film in these samples. The “staircase” offset in the chain ends explains the loss of  packing, similarly to the nematic phases in *n-*alkanes. Weak  spots were observed in a single example of a “chains-flat” C_30_H_61_OH crystal (Figure S9). The simulated patterns for “chains-flat” for the *P2*/*c* and *P2*_1_/*c* unit cells both suggest the presence of  spots as these unit cells have a higher degree of symmetry than seen in the *C2/c* unit cell. As with the alkane, it may be the benzoic acid templating induces a different molecular packing arrangement to solution crystallization. Alternatively, the lack of  spots in most alcohol SAED patterns may be due to disordered chain packing in the form of nematic phases derived from the *P2*/*c* and *P2*_1_/*c* unit cells (as opposed to ordered staircase packing). This is particularly true given that  spots were not universally observed in “chains-flat” C_31_H_64_ crystals either, despite all unit cell descriptions establishing lamellar packing as the preferred molecular packing arrangement in the *n*-alkanes.

Lines of diffuse scattering along  for  (perpendicular to row of  spots) were observed in both the C_31_H_64_ and C_30_H_61_OH SAED patterns. This diffuse scattering intensity is consistent with observations previously reported on other *n*-alkanes by Dorset.^54^ This diffuse scattering is absent in kinematical diffraction simulations of unit cell models as it can be broadly attributed to disorder. We pursue this question further below *(Microstructure in Single-Component Waxes)*.

**Figure 5:**
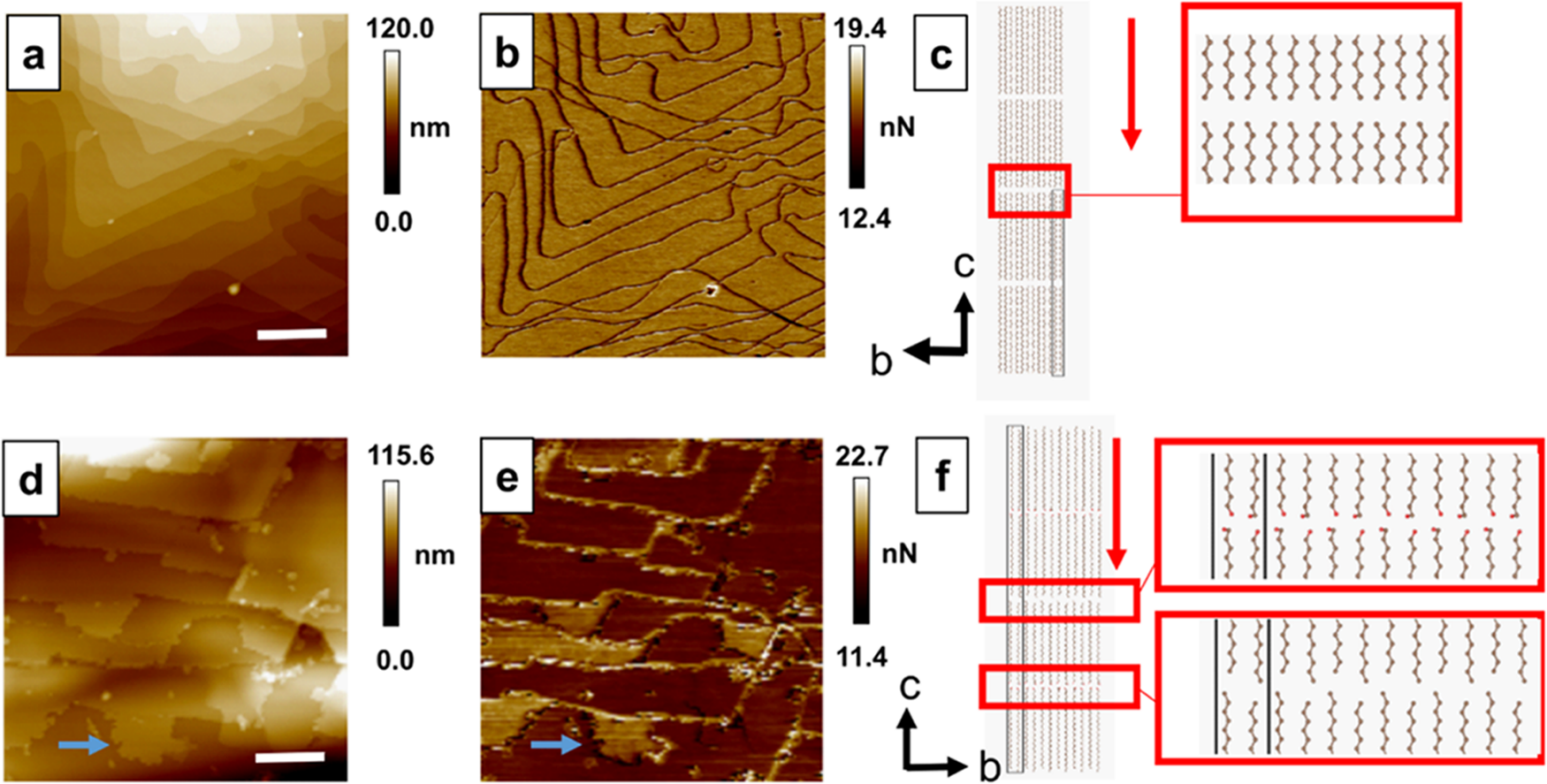
Height (a, d) and adhesion profiles (b, e) obtained through AFM for “chains-down” C_31_H_64_ (a–c) and C_30_H_61_OH (d–f) crystals. Blue arrows indicate a region in the C_30_H_61_OH crystal where a height change corresponds to an adhesion change in their respective plots. “Chains-down” unit cell representations of C_31_H_64_ (c) and C_30_H_61_OH (f) chains with a red arrow to indicate the viewing direction of the AFM probe. Magnified sections show the differences in end-to-end chain packing in the alkane vs alcohol. Scale bars indicate 1 μm.

To further probe the unit cell structural models for C_31_H_64_ and C_30_H_61_OH, we carried out atomic force microscopy (AFM) on each single-component crystal. AFM allows the measurement of surface forces such as adhesion by measuring the force between the tip attached to the cantilever and the said surface.^55^Figure 5 presents height and adhesion images obtained for C_31_H_64_ and C_30_H_61_OH crystals. Terracing, defined by plateaus with equal height steps, can be seen in both samples. These AFM images of C_31_H_64_ crystals also confirmed that crystals formed in the “down-chain” orientation exhibited well-defined facets with facet angles of ∼109° (Figure S10), consistent with BF-TEM images. The regular terracing in the C_31_H_64_ sample, enabled the construction of a height histogram (Figure S11), from which the height between steps was determined to be 4.0 ± 0.1 nm. From the crystal structure, the half-cell distance is *c*/2 = 4.1 nm, including interlamellar space, and the carbon-to-carbon distance is C_31_ – C_1_ = 3.8 nm, not including terminal protons. The AFM step height is therefore consistent with the molecular chain length extended in the expected linear conformation for C_31_H_64_.

Contrastingly, adhesion mapping of C_31_H_64_ and C_30_H_61_OH show pronounced differences between the alkane and the alcohol. C_31_H_64_ crystals exhibit a consistent, flat adhesion value across the detected terrace structures, with reduced adhesion at step edges due to the lower tip–sample contact area in regions of high curvature (Figure 5b). C_30_H_61_OH crystals, however, show patches of two different adhesion values across many terrace structures (Figure 5e). These regions of higher and lower adhesion coincide with step changes in height values. Additional profiles showing the alignment of step height and adhesion changes are shown in Figure S12. Across this field of view, C_30_H_61_OH crystals also show an average 8.3 nm step height, equal to two C_30_H_61_OH molecular chains end to end in contrast to the single-molecule step heights observed in C_31_H_64_ crystals. The height of two chains stacked in the idealized *P2*_1_/*c* structure is 8.3 nm. Notably, the height of two chains in the *C2*/*c* structure is only 6.6 nm due to the tilt of the chains, suggesting the chains do not pack with *C2*/*c* symmetry when crystallized from solution onto a flat surface.

Together, differences in adhesion corresponding to terraces and a preference for double-steps map to key features of the β-form C_30_H_61_OH unit cells (*P2*_1_/*c* and *P2*/*c*). The β-form unit cells exhibit a chemically distinct two-molecule repeat, where surfaces terminated by alcohol groups are expected to exhibit different adhesion properties to methyl-terminated surfaces. The strong hydrogen bonding between alcohol groups, with hydrogen bond donor–acceptor pairs represented particularly in the *P2*_1_/*c* structure, explains these double-step height features. Given the thermodynamically favorable hydrogen bonding interactions, unpaired or dangling alcohol groups are likely to make a significant contribution to raising the surface free energy or promoting further molecular attachment. This increases growth velocities, in comparison to methyl-terminated, van der Waals interacting surfaces. As such, double steps between methyl-terminated terraces appears the most likely step arrangement. These AFM observations, together with TEM and SAED analyses, provide experimental confirmation of end-to-end functional group interactions that distinguish C_30_H_61_OH crystals from C_31_H_64_ crystals despite otherwise similar chain lengths and polyethylene-like side-by-side chain packing.

### Microstructure in Single-Component Waxes

To set up spatially resolved, SED measurements of the native state in these beam sensitive molecular crystals, we first established their critical fluences, *C*_F_. The *C*_F_ is defined as the characteristic rate of exponential decay of a signal on exposure to ionizing radiation, such as high energy electron beams. *C*_F_ values determined from SAED at 300 kV were 6 e^–^/Å^2^ for C_31_H_64_ and for C_30_H_61_OH (Table S1). Uncertainties estimated from replicate measurements are reported in Table S2 with standard errors generally below 1 e^–^/Å^2^ with the exception of {020} reflections in C_30_H_61_OH. These appear at higher scattering angle than {110} and {200} reflections; reflections at higher scattering angles decay more rapidly and therefore contribute fewer data points for a fixed flux. These critical fluence values are in line with those previously reported values for paraffin crystals.^39^Figure S13 shows examples of Bragg spot intensity decay following an exponential, either from first electron exposure or following some initial reorientation on the grid.^56^

*C*_F_ values determined from SED were 15.1 e^–^/Å^2^ for C_31_H_64_ and 10.9 e^–^/Å^2^ for C_30_H_61_OH (Table S1), likely underestimated as the probe size (3 nm) was smaller than the pixel size (>3.8 nm). The effect of exposure in SED also depends on the distribution of intensity within the probe.^57^ We also observed mass loss, recorded as lost intensity within the exposed region in ADF-STEM imaging (Figure S14), and we therefore further determined *C*_F_ values for virtual ADF-STEM images (Figure S15) as ∼220–250 e^–^/Å^2^ for C_31_H_64_ and for C_30_H_61_OH (Table S1). Mass loss has been observed previously in molecular systems where radiolysis results in volatile products.^38^ The *C*_F_ for mass loss, observed here as an order of magnitude greater than for diffraction intensity decay, is consistent with prior work^58^ and ascribed to a damage mechanism where disruption of crystalline packing precedes volatilization of molecular fragments arising from radical reactions under the beam. Cumulatively, we confirm low-dose SED measurements at ∼10 e^–^/Å^2^ retrieve high quality diffraction signals from C_31_H_64_ and for C_30_H_61_OH crystals.

**Figure 6:**
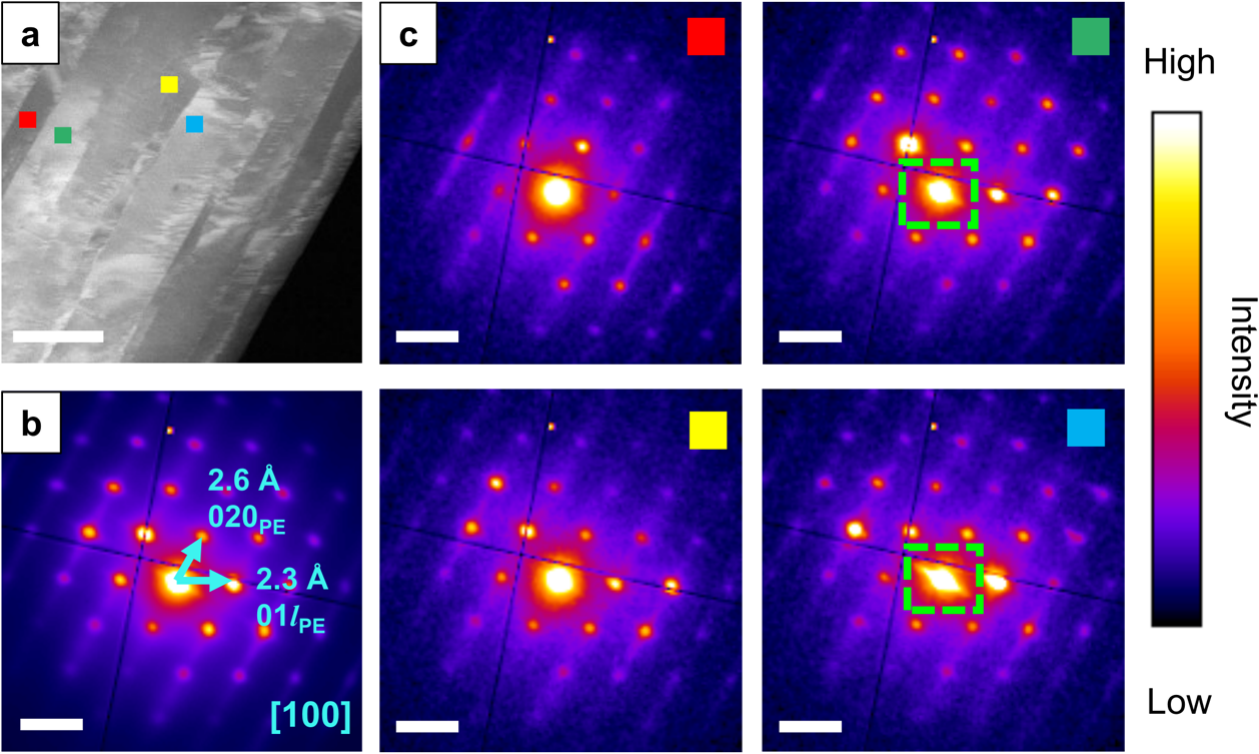
(a) ADF-STEM image of a C_31_H_64_ crystal. (b) The average diffraction pattern from the entire field of view. (c) Summed diffraction patterns from an area of 10 × 10 pixels shown by the boxes in the [100] orientated C_31_H_64_ crystal(s) (a). Dashed green rectangles indicate the presence of  spots (elongated intensity around the zero order spot running diagonally top left to bottom right). The ADF-STEM image scale bar indicates 500 nm. All diffraction pattern scale bars indicate 0.5 Å^–1^.

In turn, we next report nanometer-resolved SED analyses of C_31_H_64_ crystals. Figure 6 shows an SED data set from C_31_H_64_ crystals prepared in the “chains-flat” orientation to examine lamellar packing. Figure 6a shows a low-angle ADF-STEM image (formed using an inner angle of 2.1 mrad and outer angle of 7.5 mrad; a visualization of the virtual detector is found in Figure S15). Figure 6b presents the corresponding average diffraction pattern from the entire field of view. The diffraction pattern was indexed to the same *A2*_1_*am* unit cell as in SAED (Figures 6 and S6). The reciprocal space c*-axis indicates the chains lie perpendicular to the long surface facets. The pattern also exhibits diffuse scattering along  for  as observed by SAED.

The ADF-STEM image exhibits significant variation in intensity, consistent within elongated rectangular regions in the image. To understand the origin of this image contrast, we extracted spatially resolved diffraction patterns from across a set of these regions (Figure 6c). These patterns show the same set of Bragg spots as in the average pattern (Figure 6b) but exhibit variation in the intensities of spots across the pattern. Figures S16 and S17 show further examples of similar observations in additional C_31_H_64_ crystals. We further confirmed this contrast variation emerges from diffraction intensity variation by constructing virtual ADF images across a series of ADF collection angles (Figure S18). With increasing collection angle beyond the Bragg scattering in the zero order Laue zone the contrast deteriorates, indicating the contrast primarily emerges from variation in diffraction intensities.

The observed Bragg spot intensity variations can be understood as arising from small variations in the local orientation of the crystal within these rectangular regions or ‘grains.’ Through comparison with kinematical simulations of mis-tilted C_31_H_64_ crystals (Figure S19), the spatially isolated patterns (Figure 6c) were assigned to orientations <1° from the [100] zone axis. The misorientation analysis revealed two angular variations, one away from the [100] axis and a further rotation about this axis, though with no rotation of the pattern on the detector itself. The variation in the second rotation about [100] follows a consistent counterclockwise rotation in the diffraction data when moving from left to right in the image (Figure S19), suggesting a degree of coherence likely emerging during crystal growth on or against adjacent grains within the larger wax crystal plate.

Figure 6c also highlights the presence of  spots observed in some, but not all, spatially resolved diffraction patterns (dashed green rectangles). These  spots are a defining characteristic of well-ordered end-to-end chain packing in lamellar domains as illustrated earlier in Figure 2. We have ruled out variation in the intensities of the  spots due to orientation changes based on comparison with electron diffraction simulations (Figure S19). Instead, the observation of  spots in only some areas of the crystal plate indicates there are variations in the order or disorder of the lamellar packing across the grains in the field of view.

**Figure 7:**
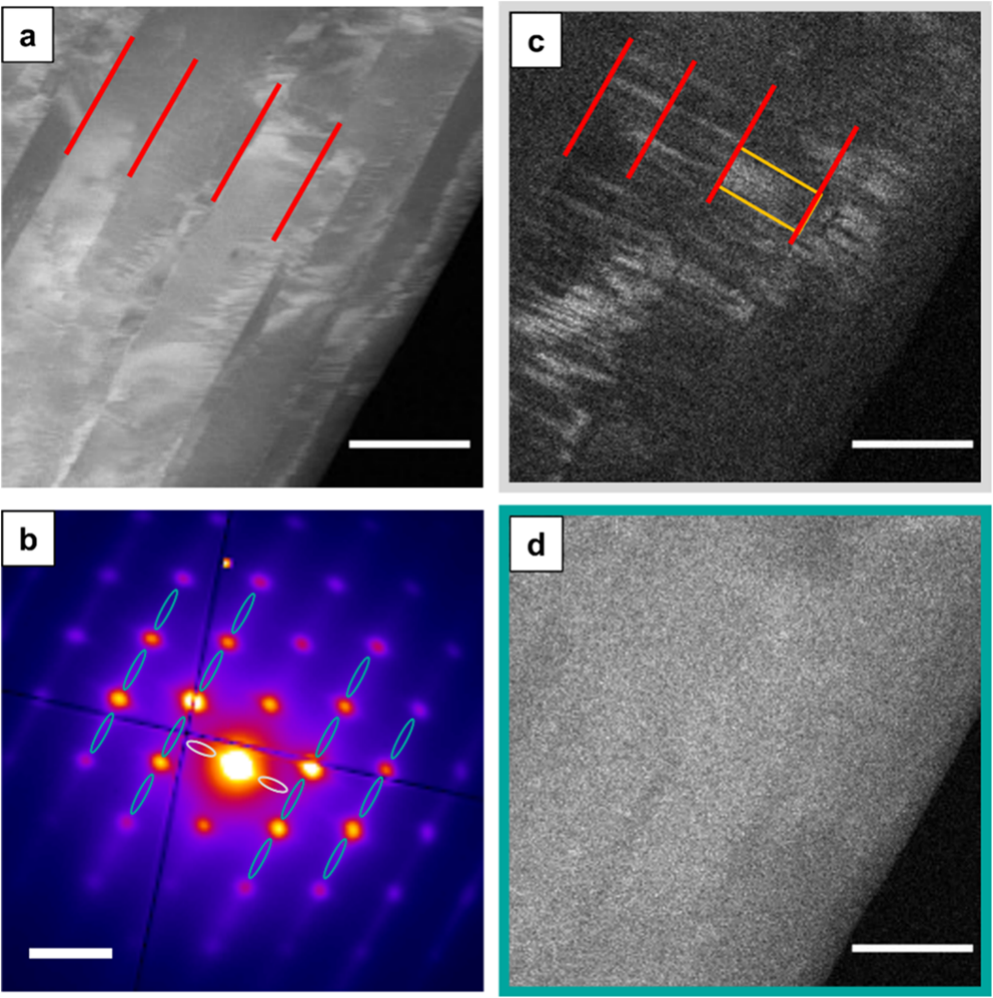
(a) Low-angle ADF image of a C_31_H_64_ epitaxially orientated crystal with grain boundaries highlighted with red lines. (b) Average diffraction pattern of crystal in (a). (b) Average diffraction pattern. (c) VDF () image produced from signal area defined by the gray ovals in (b), with grain boundaries highlighted with red lines and a domain of lamellar ordering highlighted by orange lines. (d) VDF image produced from diffuse scattering signal in the diffraction pattern defined by the blue ovals in (b). All STEM image scale bars indicate 500 nm. The diffraction pattern scale bar indicates 0.5 Å^–1^.

To isolate the spatial variation in  spot intensity, we constructed a virtual dark field (VDF) image formed from the  spots by placing small virtual apertures around these features, effectively as a postprocessing mask on the diffraction plane in the four-dimensional data set (shown on the average diffraction pattern, Figure 7). The placement of the virtual apertures is shown in Figure 7b, and the  corresponding VDF image is shown in Figure 7c. Narrow rectangular domains of bright intensity appear in the  VDF (Figure 7c), providing a map of the location of ordered lamellae within the C_31_H_64_ grains.

To further confirm the domain assignment of lamellar ordering of the  VDF intensity, we extracted diffraction patterns only from bright regions in the  VDF (Figure S20). Ordered lamellae are expected to exhibit distinct diffraction intensities both at  and the  first order reflections for  (Figure 2), with the  as the single, dominant feature when no lamellar order is retained (nematic phases). While the SED patterns do not completely resolve the  or  spots, the spots show defined elongation along  and . These two features are correlated in spatially resolved diffraction patterns from across the field of view (Figure S21) with increases in  intensity corresponding to increased ellipticity at the  first order reflections. The lower SED pattern resolution, relative to SAED patterns in Figure 4, arises directly from a trade-off in spatial resolution and momentum transfer resolution as the convergence semiangle of the probe is set to provide a nanoscale probe (<1 mrad) which in turn gives rise to overlap between finely spaced reflections (appearing as disks with radius equal to the convergence semiangle) in the diffraction pattern. As such, we apply both SAED and SED for crystallographic phase assignment and grain and domain analysis, respectively.

The long axis of the domains of  intensity lies perpendicular to the major surface facet and is therefore aligned with the row of  spots and the c*-axis. That is, the long axis of the domains aligns with the molecular chain axis. The average length of these domains determined across six fields of view was 332 nm (sample standard deviation *s* = 112 nm). For *c* = 8.26 nm, these lines correspond to approximately 40 unit cells packing together, i.e., 80 molecules packing end to end (*s* ∼ 14 unit cells). These standard deviations refer to the width of the distribution of lengths rather than an uncertainty in the precision of their measurement.

While in several cases the  domains extend across the entire width of the grain they are part of, the domains are not all the full width of the grains. Moreover, there is a clear break in the intensity between  domains at the grain boundaries. These observations point to local, highly ordered end-to-end packing that is confined within a grain. The ordered end-to-end packing may also extend only part way across the grain, indicating a transition to nematic phases within the width of single grains.

The SED data also enable the separate analysis of the  intensity and the lines of diffuse intensity at  at fixed . Figure 7d shows a VDF image formed from these regions of diffuse scattering. This VDF image shows even intensity across the entire field of view, indicating no correlation between the end-to-end chain ordering and the diffuse scattering intensity. We note that this image in fact contains isotropic diffuse background scattering (varying with thickness) as well as the anisotropic diffuse scattering streaks. The diffuse scattering streaks are, however, significantly above the isotropic background, and when an isotropic background is subtracted the diffuse scattering streak signal likewise shows a homogeneous distribution in the crystal (Figure S22 and Table S3). Figure S23 presents further inspection of the diffuse scattering intensity across different grains. These differences in the mapped VDF intensities identify distinct structural origins for the disorder contributing to the diffuse scattering intensity and the disorder contributing to the loss of  spot intensity.

**Figure 8:**
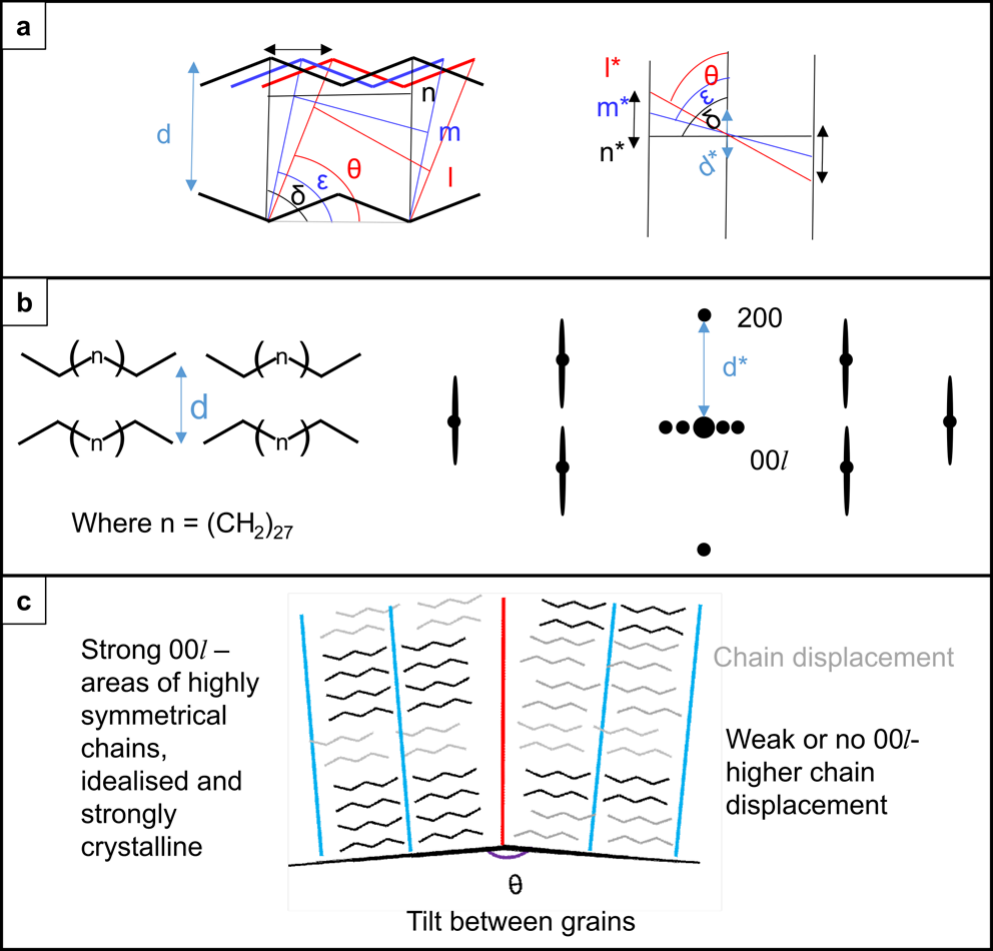
(a) Proposed model to explain how chain offset may contribute to diffuse scattering in the observed diffraction patterns, adapted from Eggeman.^50^ (b) Simplified representation of the unit cell of the C_31_H_64_ packing looking down the [100] direction and corresponding diffraction pattern formed from this chain orientation. (c) Proposed structural model to show how chain packing and offset contributes to the diffuse scattering and  spots seen. Chain packing on the left and right of the red line correspond to the grains seen, and the tilt seen between them. The left grain corresponds to one with more domains of ordered packing and therefore higher intensity Bragg diffraction. The right grain corresponds to one of higher chain displacement and therefore lower intensity Bragg diffraction.

Figure 8 presents a unified structural model for each of the observed diffraction signals. Beginning with the diffuse scattering, Figure 8a depicts displacements along the chain axis that give rise to diffuse scattering streaks in the diffraction pattern perpendicular to the chain axis. This schematic follows established models for dynamical disorder in molecular crystals exhibiting linear displacements, e.g., due to vibrational modes.^50^ The linear displacement of the molecule creates a range of interplanar angles (δ, ε, θ) and interplanar spacings (*l*, *m*, *n*) that contribute to a line of diffuse diffraction intensity reflecting the distribution of chain displacements. We have additionally built a model to illustrate how chain displacement in this structure contributes to the diffuse scattering signal. Figure S24 shows a simple model where one chain has been displaced in a supercell constructed from 10 repeats of the C_31_H_64_*A2*_1_*am* unit cell. Figure S24c shows the resultant simulated diffraction pattern where a displacement has been applied for a range of supercell sizes as a simple model of a distribution of displacement periodicities as expected in dynamical disorder. The presence of diffuse scattering streaks in this model (for reflections  where ) illustrates how unidirectional chain displacement contributes to the diffuse scattering signal observed experimentally. A simplified representation of C_31_H_64_ chain orientations viewed along [100] and the corresponding diffraction pattern are shown in Figure 8b to illustrate how this model relates to the real space packing of the chains and to the resulting diffraction pattern. While the SED data do not distinguish between static and dynamical disorder, the absence of correlation with the loss of  intensity points to dynamical displacements. Previous work on waxes has likewise shown a decrease in diffuse scattering in SAED when cooled,^59^ suggesting that the displacements likely arise from dynamical disorder.

Figure 8c in turn combines the diffuse scattering model with the grain and domain structures recorded in SED. Across the largest length scales, C_31_H_64_ grains exhibit small relative mis-orientations of up to ∼1°, forming a microstructure comprising elongated rectangular grains micrometers in length and <500 nm in width. Within these grains, there are well-ordered lamellar domains exhibiting strong  diffraction forming anisotropic domains ∼300 nm long and ∼40 nm wide as well as regions where nematic ordering dominates. Within both well-ordered lamellar domains and disordered nematic phases there is additionally dynamical disorder along the chain axis. Beyond a simple “brick and mortar” model, our description identifies regions of lamellar order coexisting with nematic phases rather than identifying fully amorphous interface regions.

**Figure 9:**
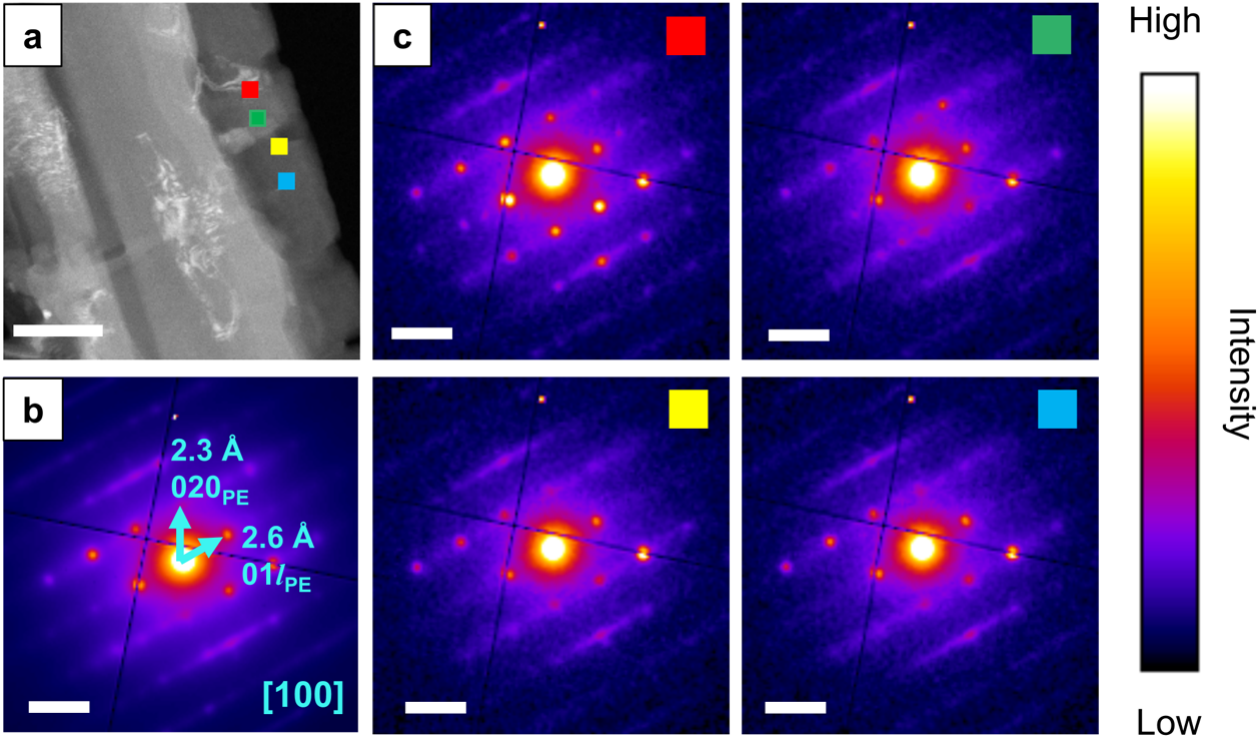
(a) ADF-STEM image of a C_30_H_61_OH crystal. (b) The average diffraction pattern from the entire field of view. (c) Summed diffraction patterns from an area of 10 × 10 pixels shown by the boxes in the [100] orientated C_30_H_61_OH crystal (a). The ADF-STEM image scale bar indicates 500 nm. All diffraction pattern scale bars indicate 0.5 Å^–1^.

A similar SED analysis was carried out on C_30_H_61_OH in the “chains-flat” orientation. Figure 9 presents a low-angle ADF-STEM image, average diffraction pattern, and spatially resolved diffraction patterns extracted from areas of equivalent size to those in Figure 6 for C_31_H_64_. Figures S25 and S26 show additional examples of similar C_30_H_61_OH crystals. These C_30_H_61_OH crystals were indexed as for SAED (Figure S6) to the [2 0 45] zone axis for a modeled *C2*/*c* unit cell for C_30_H_61_OH. The C_30_H_61_OH crystals likewise exhibit diffuse scattering as for C_31_H_64_ indicating a similar dynamical chain displacement disorder mode in the alcohol and in the alkane. Figure S27 presents analyses of the diffuse scattering intensity across additional grains.

Both the low-angle ADF-STEM image and the corresponding diffraction patterns show distinct differences to the features observed in C_31_H_64_ crystals (Figures 6, 9). First, no grain structure is observed in the low-angle ADF-STEM image of C_30_H_61_OH. Overall, the crystal exhibits a blocky structure as seen in TEM (Figure 4), but grain structure is not observed within these blocks in either TEM or SED. Where there are variations in contrast, inspection of diffraction patterns indicate that these arise from mass–thickness contrast (Figures S28, S29). Spatially isolated diffraction from similar length scales to the grain structure in C_31_H_64_ show no changes in the distribution of diffraction intensity (Figure 9). Second, no  diffraction spots or signatures of elliptical elongation of spots were observed in any C_30_H_61_OH crystals. These observations are consistent with simulations of the γ-form *C2*/*c* unit cell containing staircase packing in the alcohol or, for the β-form unit cells (*P2*_1_/*c* or *P2*/*c)*, nematic phases (Figure S6). We additionally formed VDF images using a virtual aperture for  spot positions, but no contrast attributable to  diffraction was observed (Figures S30–S32).

The staircase or nematic phase packing as well as the strong hydrogen bonding interactions between paired alcohol groups within C_30_H_61_OH crystals (Figures 1–3, S33) likely explains the observed differences in crystal formation in the “chains-flat” orientation between C_31_H_64_ and C_30_H_61_OH. Grain formation is likely disfavored by the less ordered stacking in the staircase or nematic phase structure. Moreover, changes in orientation across alcohol-terminated groups requires distortion and loss of stabilizing hydrogen bonding interactions (Figure S33). Together, the presence or absence of grain microstructure and lamellar ordering stand out as defining characteristics of each endmember (C_31_H_64_ and C_30_H_61_OH), setting up further examination of intermediate compositions in binary mixtures.

### Binary Mixtures

**Figure 10:**
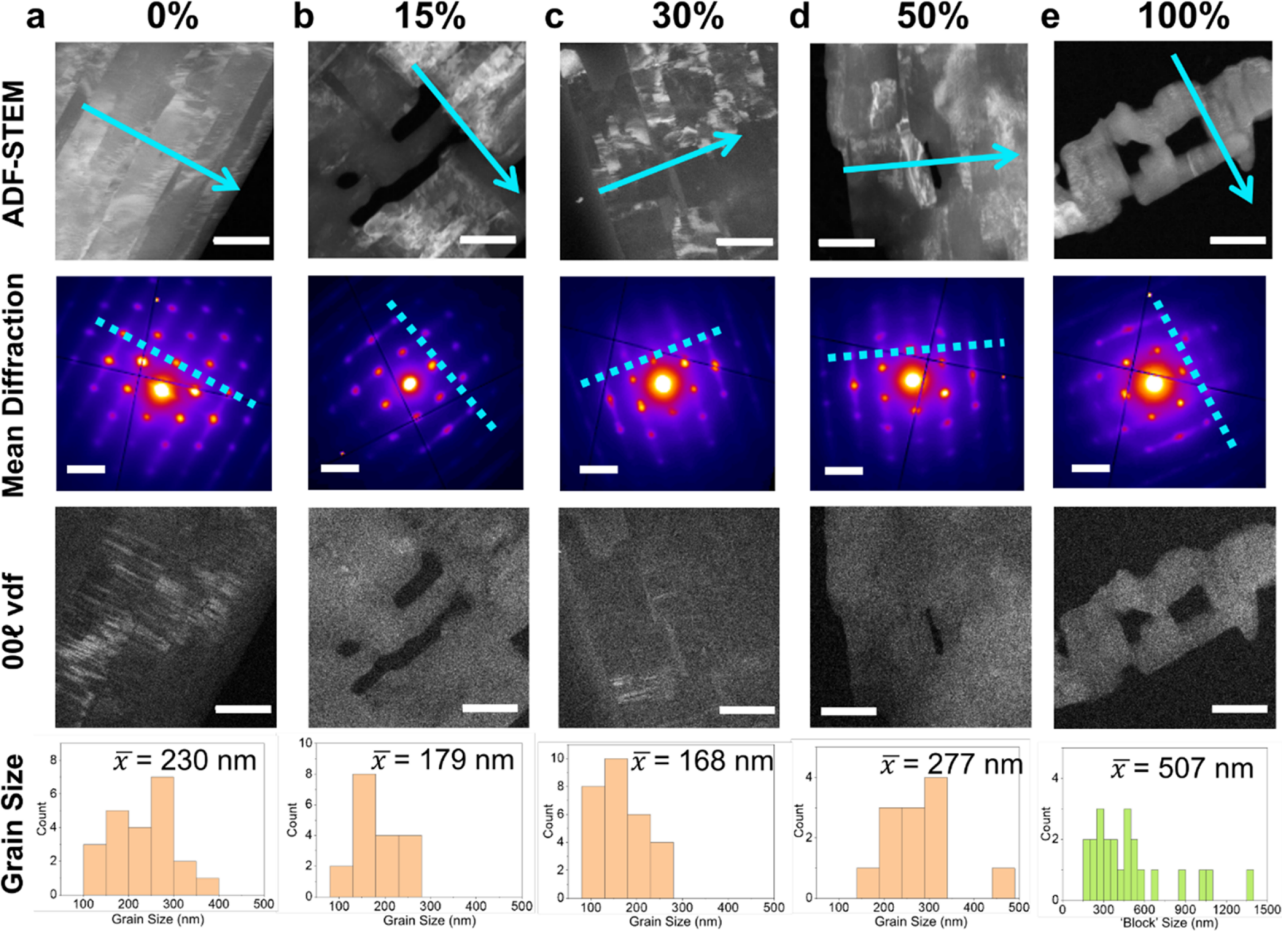
ADF-STEM images and corresponding average diffraction patterns of [100] orientated binary mixture crystals. Compositions range from (a) pure phase C_31_H_64_, (b) 15%, (c) 30%, and (d) 50% C_30_H_61_OH (increasing alcohol content) to (e) pure phase C_30_H_61_OH. The percentage indicates the content of alcohol by molar fraction in the composition. Green arrows indicate the *c*-axis direction (alkyl chain axis) following the polyethylene cell (*c*_PE_). Arrows overlaid on the SAED patterns mark the indexation of the patterns to the PE cell in the [001]_PE_ “down-chain” orientation and the [100]_PE_ “chains-flat” orientation. Corresponding VDF images, produced from the signal area in the average diffraction pattern for each corresponding data set along the c* direction next to the central beam (where a  line signal should arise), are found in the third row. Histograms of measured grain sizes for each respective composition and the average grain size are included below. The histogram below the pure phase C_30_H_61_OH ADF image and diffraction pattern indicates the measured “block” sizes, believed to indicate the size of extended crystal growth in this system. All ADF-STEM image scale bars indicate 500 nm. All diffraction pattern scale bars indicate 0.5 Å^–1^.

Figure 10 extends the SED analysis to binary mixtures from 15% to 50% C_30_H_61_OH with C_31_H_64_. This range corresponds to the compositions of biological interest, with the total alcohol content estimated at 15–30% in the *S. elegantissima* plant.^31^Figure 10 shows low-angle ADF-STEM images and corresponding average diffraction patterns from the entire field of view for each endmember as well as for 15%, 30%, and 50% C_30_H_61_OH. The low-angle ADF-STEM images reveal defined grain microstructure across compositions from 0–30% C_30_H_61_OH, with only some grain structure visible at 50%. The grain structure, where visible, follows a consistent crystallographic orientation with the short axis of the grains aligned with the unit cell *c*-axis (Figure 10, dashed and solid blue lines in diffraction and ADF-STEM images, respectively). Overall, with increasing C_30_H_61_OH content the definition of grain boundaries decreases, with shorter and more curved boundaries appearing at 30% and 50% C_30_H_61_OH. These observations may reflect increasing nematic phases (tending toward staircase-like packing) bridging across grains. We formed VDF images using a virtual aperture for  spot positions for 15%, 30%, and 50% C_30_H_61_OH in addition to the VDF images formed for the pure endmembers (Figure 10, third row). No contrast attributable to  diffraction was observed in the 50% C_30_H_61_OH VDF image. Some bright patches were seen in the 15% C_30_H_61_OH VDF image, whereas some bright sharp lines of intensity, as seen in the pure C_31_H_64_ VDF image, were seen in the 30% C_30_H_61_OH VDF image. Again, to further confirm the domain assignment of lamellar ordering of the  VDF intensity, we extracted diffraction patterns only from bright regions in the  VDF for both 15% and 30% C_30_H_61_OH (Figures S34 and S35). The presence of  spots in these diffraction patterns, not seen in the average diffraction pattern, confirms some lamellar order within the crystals. This ordering is not to the same extent as we see in the pure C_31_H_64_, demonstrating disruption to the lamellae on addition of alcohol to the binary mixture.

Histograms of grain size, taken as the grain width, showed no consistent trend across this composition range (Figure 10, bottom row). The average grain size decreased from 0% to 15% to 30% C_30_H_61_OH, but falls broadly between 150–300 nm. Certainly the incorporation of moderate quantities of C_30_H_61_OH does not alter the grain size substantially. Notably, the increase in average grain size at 50% C_30_H_61_OH may arise from reduced precision in measurements due to the loss of defined boundaries. As no grains were visible in 100% C_30_H_61_OH, a “block” size was measured instead to evaluate any similarity in these blocks and the grains observed in the other compositions. The average size of these blocks in 100% C_30_H_61_OH was 507 nm, roughly double the size of the grains seen in the pure C_31_H_64_ and binary mixtures, and so we ruled out any correspondence between these features and the tilted grains in 0–50% C_30_H_61_OH.

**Figure 11:**
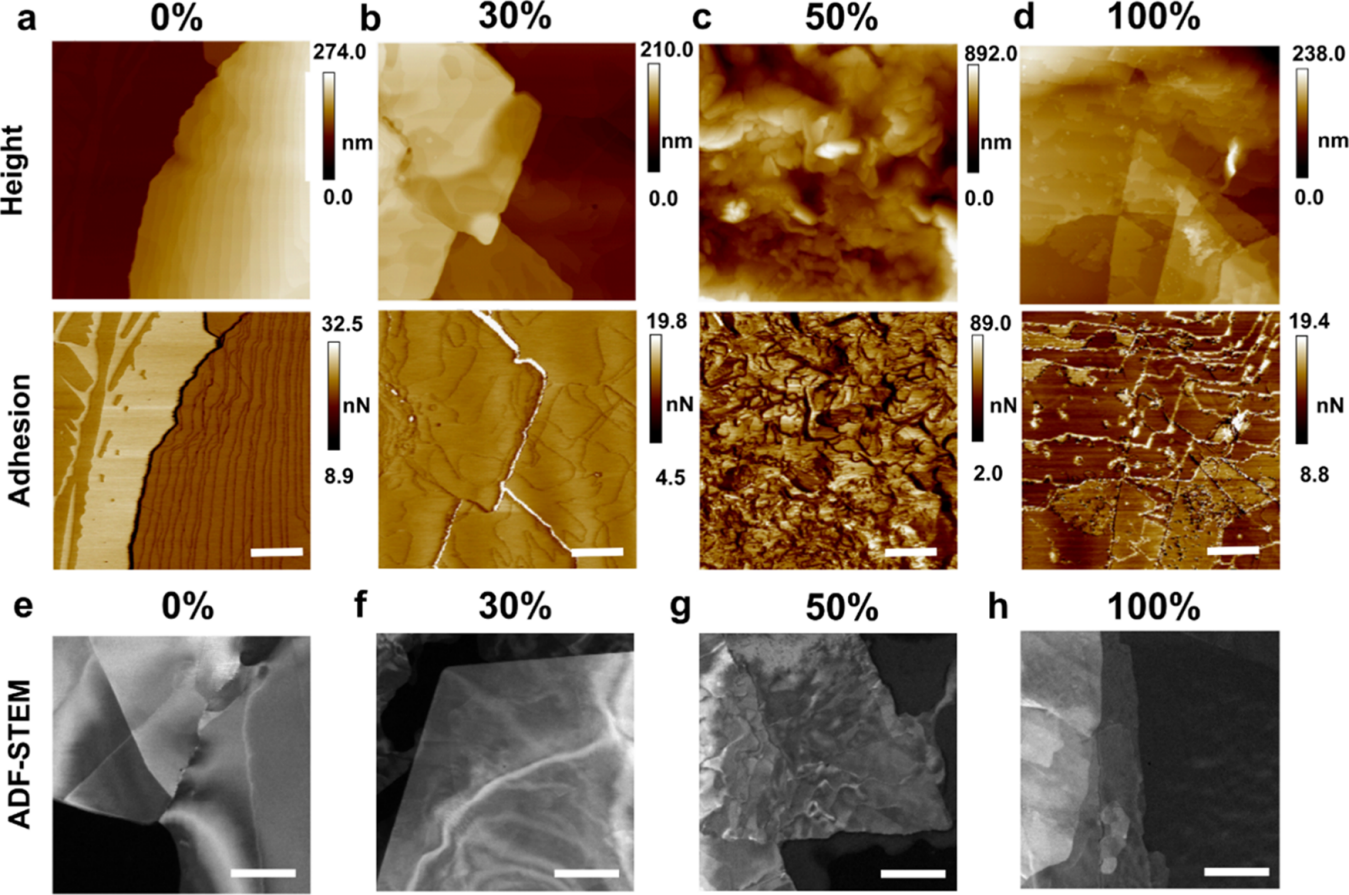
Height and adhesion profiles (a–d) obtained through AFM, and ADF-STEM images (e–h) for pure phase alkane, alcohol and binary mixture samples. Compositions range from pure phase C_31_H_64_ (a, e), with increasing alcohol content, to pure phase C_30_H_61_OH (d, h). The percentage indicates the content of alcohol by molar fraction in the composition. The two AFM panels in the 100% alcohol composition column are lower magnification scans of the sample shown in Figure 5. Scale bars for the AFM data indicate 2 μm. Scale bars for the STEM images indicate 500 nm.

Recognizing the value of adhesion mapping by AFM for the observation of alcohol group termination in the “down-chain” orientation (Figure 5), we acquired further AFM data for the binary mixtures in the “down-chain” orientation. Figure 11 presents height and adhesion mapping by AFM of 0%, 30%, 50%, and 100% C_30_H_61_OH prepared by drop-casting on mica as well as matched low-angle ADF-STEM images from SED prepared by drop-casting on to continuous carbon TEM grids. For 0 and 30% C_30_H_61_OH, the images all show faceted crystals with terraces (height images) with consistent adhesion. The low-angle ADF-STEM images exhibit some bend contour contrast but otherwise show smoothly varying contrast within micron-sized regions. At 50%, both AFM and low-angle ADF-STEM highlight pronounced differences: The AFM height image shows a much more substantial variation in height at the submicrometer length scale. As with the pure alcohol, the adhesion values at 50% C_30_H_61_OH are bimodal, seen more clearly in Figure S36. The adhesion image likewise exhibits submicrometer variations, and contrast variations with irregular contours at similar length scales were also recorded in low-angle ADF-STEM. At 100% C_30_H_61_OH, the AFM height once again was comprised of terraces with the characteristic bimodal adhesion variation between steps. The low-angle ADF-STEM image for C_30_H_61_OH also shows faceted crystals with micrometer-scale limited intensity variation within plates.

The lack of bimodal adhesion variation between steps at 30% C_30_H_61_OH shows no observable systematic ordering of the terminal alcohol groups at this composition. At 50% C_30_H_61_OH, the adhesion data together with significant contrast variations in low-angle ADF-STEM may point to separation of phases in these drop-cast mixtures (Figure 11), possibly into two or more immiscible phases. Up to 30% C_30_H_61_OH, the data appears consistent with the formation of a single crystal, likely a solid solution within C_31_H_64_, as may be expected from the rich solid solution formation expected from alkanes of similar lengths.^11,60,61^ SED of the “chains-flat” samples point to a similar conclusion, with the loss of distinct grain structures arising above the 30% C_30_H_61_OH (Figure 10). At higher C_30_H_61_OH content, the competition between staircase packing in C_30_H_61_OH and lamellar ordering in C_31_H_64_ likely introduces structural disruption of the wax microstructure. Due to the similarity in *d*-spacings, however, an unambiguous evaluation of changes in the crystal phases with compositions beyond the systematic changes in disordered microstructure is not possible by these electron diffraction measurements.

### Implications for Leaf Waxes and Related Materials

The central role of the IW layers on plant leaves in controlling functions such as in restricting water loss and determining molecular diffusion rates to the leaf surface is well established and is important for both biological understanding and for developing agrochemical crop protection strategies.^5,62^ Directly observing and quantifying the heterogeneous “brick and mortar” model, the hypothesized leaf wax structure, is therefore crucial for understanding the mechanisms underpinning its functions. Our nanoscale analyses of replica leaf waxes reveal the coexistence of highly ordered domains within larger grains within each crystal together with nematic phases of disordered chains surrounding the domains of lamellar packing. These observations refine the “brick and mortar” model with nanoscale crystallographic precision. Moreover, the systematic variation in nematic phase content, loss of lamellar domains, and reduction in grain microstructure with alcohol content points to the balance of *n*-alkane and *n*-alkanol composition as a source of composition-determined microscopic diffusion pathways through the IW layer in plant leaves. The microscopic heterogeneity of these waxes provides the structural outlines for mechanistic models of layer flexibility and permeability (to water) in leaf waxes. While we focus primarily on the application of this work to understanding the microstructural heterogeneity in leaf waxes, these findings offer methods as well as structural features relevant for consideration in other related materials systems. Such systems include wax-tuned lipid crystallization pathways in oil in water emulsions in food and pharmaceutical applications^63^ and pharmaceutically relevant formulations such as layered structures, e.g., in the form of coatings^64^ or layered nanoparticles,^65^ or formulations containing long chain polymer systems, e.g., solid dispersions,^66^ where product performance is based on interactions between multiple, distinct microscopic structures.

## Conclusions

Long chain alkane (C_31_H_64_) and alcohol (C_30_H_61_OH) crystals relevant to the composition of IW layers in plant leaves have been prepared in two orientations for the examination of crystals along and across the chain axis by electron and atomic force microscopy. The orientation and possible unit cell assignments were evaluated using SAED. AFM height and adhesion mapping showed alternate packing of terminal alcohol groups in C_30_H_61_OH. Low-dose SED revealed a hierarchical tilted grain microstructure in C_31_H_64_, containing ordered lamellar domains as well as nematic phases and dynamical disorder along the chain axis. Grain microstructure was absent in C_30_H_61_OH crystals and no lamellar ordering was observed. These observations suggest significant staggered or disordered alignment of chain ends as described in nematic phase packing, possible in β-form C_30_H_61_OH, or ordered staircase packing typical of γ-form C_30_H_61_OH. Experimental evidence of grain microstructure, mis-aligned chain ends, and nematic phases coexisting with lamellar ordering and dynamical disorder suggest the unit cell descriptions serve as guideposts only for the “endmember” structural motifs in what is otherwise a complex, nanoscale landscape comprising a distribution of structures at the nanoscale.

Moreover, not only does this heterogeneity of ordered and disordered structures vary spatially, it also varies systematically with composition in binary mixtures of these *n*-alkanes and *n*-alkanols. At intermediate compositions, increasing alcohol content correlated with a loss of grain structure in “chains-flat” crystals and changes toward smaller, granular structures in “down-chain” crystals. Our microscopic observations separate and assign features not otherwise distinguishable in measurements at lower spatial resolution. Disentangling lamellar ordering and diffuse scattering at the nanoscale opens new avenues for analysis of diverse wax crystals and to test order–disorder hypotheses only indirectly assessed previously.

The retention of microstructure reminiscent of the single-component C_31_H_64_ wax up to 30% C_30_H_61_OH suggests likely solid solution behavior in the composition range of plant IW layers. The presence of regions with nematic ordering mirror the “mortar” description in the prevailing “brick and mortar” structural hypothesis, i.e., the introduction of C_30_H_61_OH may provide the disordering required for high-diffusion pathways. Compositions at or beyond 50% C_30_H_61_OH appear to introduce more significant microstructural modifications. These findings establish an initial basis for examining a range of chain tilts within a lamella, more complex wax compositions, and the interaction between functional groups, microstructure, and wax materials properties.

## Supporting Information Available

Additional visualizations of crystal structure models and the SED technique, supporting electron microscopy and atomic force microscopy analyses, and diffraction pattern simulations (PDF)
Structural Models (ZIP)
