# Supplementary material for: Grain and Domain Microstructure in Long Chain N-Alkane and N-Alkanol Wax Crystals
Source: Cryst Growth Des. 2024 Dec 7;24(24):10127–42. doi: 10.1021/acs.cgd.4c00909 (PMC11660156; doi:10.1021/acs.cgd.4c00909)
Supplement: Supplementary file 1 — cg4c00909_si_001.pdf [file cg4c00909_si_001.pdf]

Supporting Information

Grain and Domain Microstructure in Long Chain *N*-Alkane and *N*-Alkanol Wax Crystals

Emily Wynne[1, 2], Simon D. Connell[2, 3], Rachael Shinebaum[4], Helen Blade[5], Neil George[1, 6], Andy Brown[1, 2] and Sean M. Collins[1, 2, 7]*.*

[1]School of Chemical and Process Engineering, University of Leeds, Woodhouse Lane, Leeds, LS2 9JT, UK.

[2]Bragg Centre for Materials Research, University of Leeds, Woodhouse Lane, Leeds, LS2 9JT, UK.

[3]School of Physics and Astronomy, University of Leeds, Woodhouse Lane, Leeds, LS2 9JT, UK.

[4] AstraZeneca, Technical Operations Science & Innovation, Pharmaceutical Technology & Development, Operations, Macclesfield, SK10 2NA, UK.

[5]AstraZeneca, Oral Product Development, Pharmaceutical Technology & Development, Operations, Macclesfield, SK10 2NA, UK.

[6]Syngenta, Jealott's Hill, Warfield, Bracknell, R42 6EY, UK.

[7]School of Chemistry, University of Leeds, Woodhouse Lane, Leeds, LS2 9JT, UK.

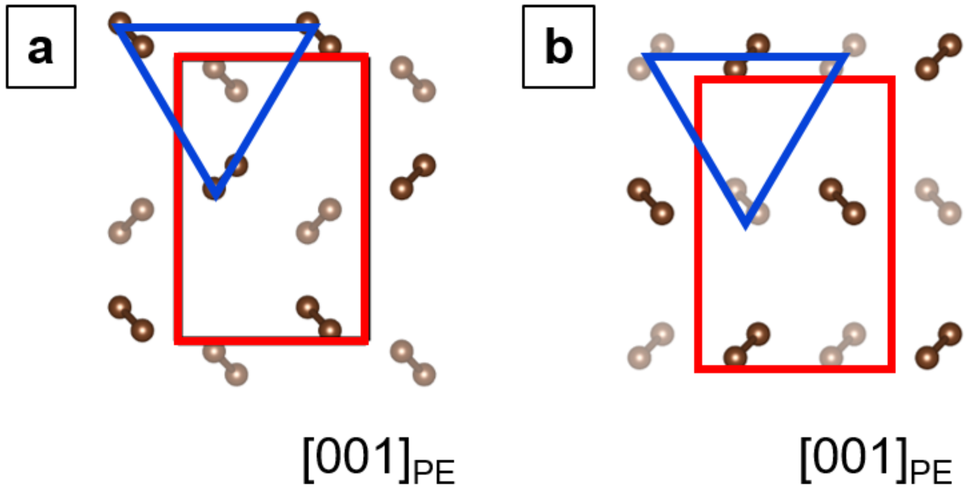

Figure S1: Representation of carbon chain packing as seen by the electron beam in the 'down-chain' orientation for the alkane packed with (a) *Pbcm* symmetry and (b) *A2₁am* symmetry. The red boxes indicate the unit cell. The blue triangles show the gap that forms between chains in neighbouring lamellar layers. Alternate chains are faded to represent the offset of chains in real space in neighboring lamellar layers.

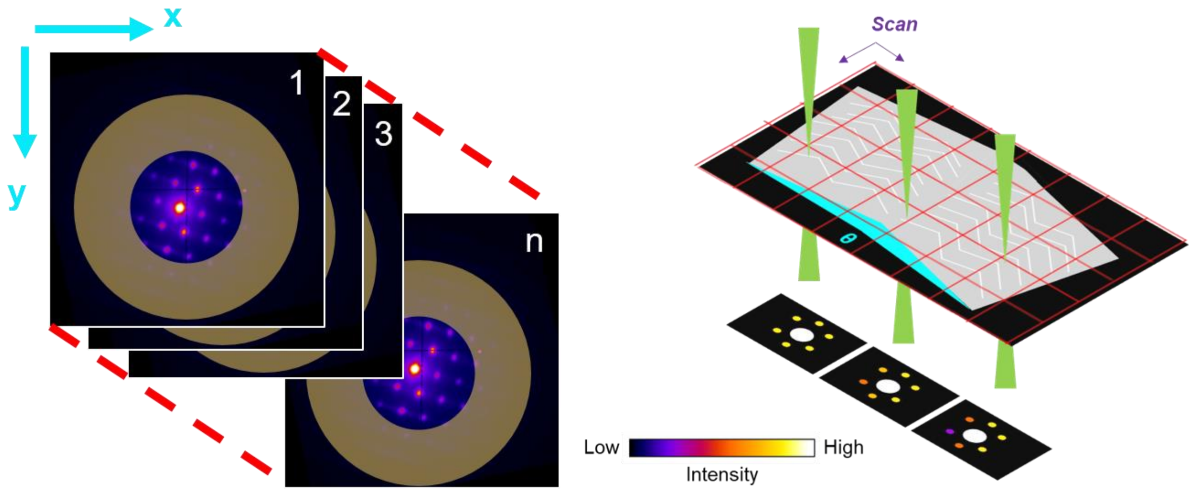

Figure S2: A simplified schematic to illustrate the 4D-STEM technique. A highly parallel probe is scanned pixel by pixel across a sample and a 2D diffraction pattern is acquired at each position up to "n" number of positions. This schematic represents the change in intensities seen in the Bragg spots in a diffraction pattern dependent on the relative tilt of the sample at each position relative to the electron beam. The outcome is a 4D dataset where both individual and average patterns can be extracted in post processing. When a virtual aperture is defined and placed, the intensities in the entire dataset are integrated within this detector region.

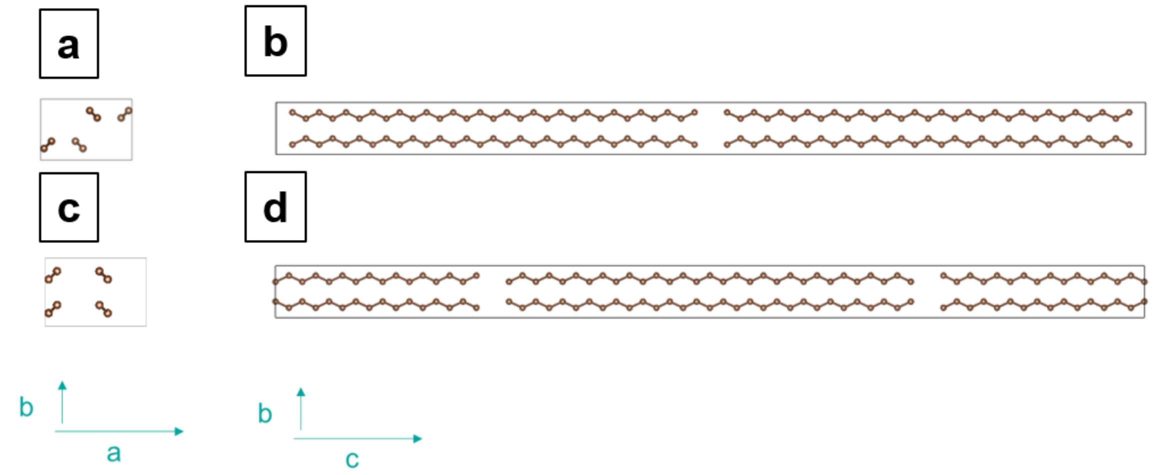

Figure S3: (a,c) Representation of carbon chain packing in $C_{31}H_{64}$ as seen by the electron beam in the 'down-chain' drop cast prepared orientation, the [001] direction for *Pbcm* (a) and $A2_1am$ (c) symmetry. (b,d) Representation of carbon chain packing in $C_{31}H_{64}$ as seen by the electron beam in the 'chains-flat' epitaxially prepared orientation, the [010] direction for *Pbcm* symmetry (b) and the [100] direction for $A2_1am$ symmetry (d). The *Pbcm* and $A2_1am$ orthorhombic unit cells are defined by a = 7.44 Å, b = 4.93 Å, c = 82.59 Å.

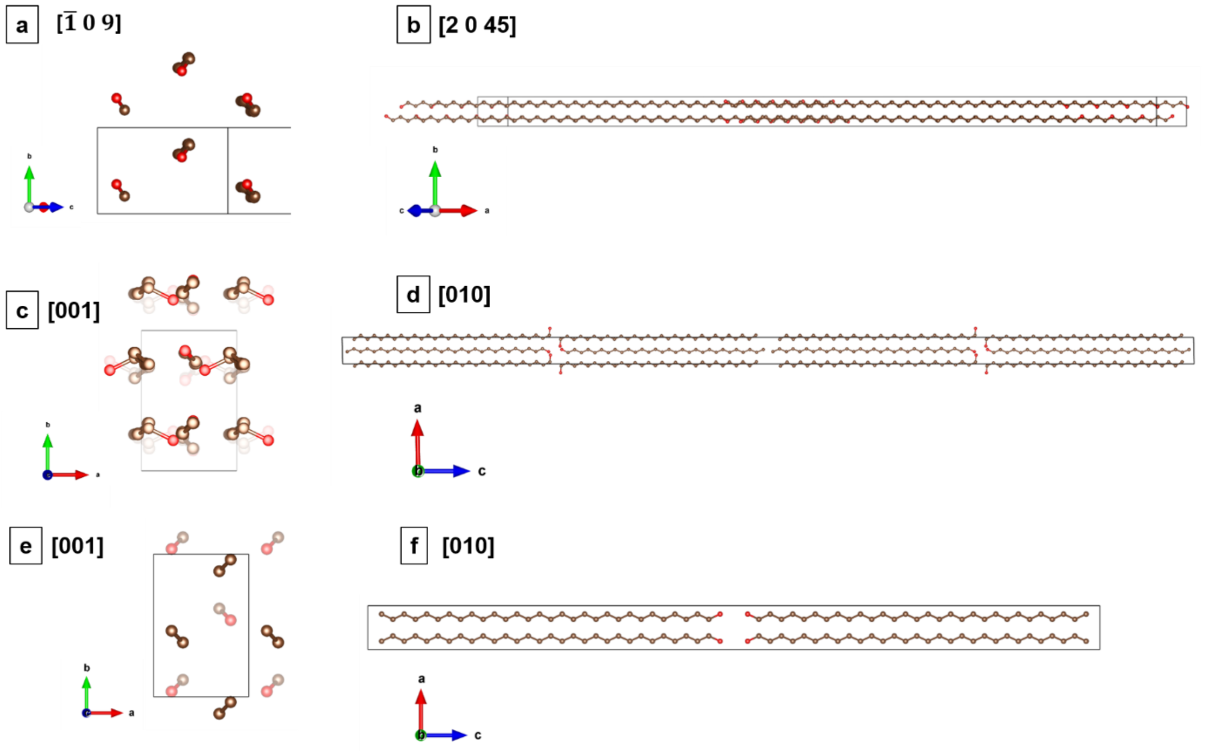

Figure S4: (a,c,e) Representation of carbon chain packing as seen by the electron beam in the 'down-chain' drop cast prepared orientation, the $[\bar{1}\ 0\ 9]$ for *C2/c* symmetry (a), and the [001] direction for *$P2_1c$* (c) and *P2/c* symmetry (e). (b,d,f) Representation of carbon chain packing as seen by the electron beam in the 'chains-flat' epitaxially prepared orientation, the [2 0 45] direction for *C2/c* symmetry (b), and the [010] direction for *$P2_1c$* (d) and *P2/c* symmetry (f). The *C2/c* monoclinic unit cell is defined by a = 132.91 Å, b = 4.93 Å, c = 9.00 Å, β = 93.01º. The *$P2_1/c$* monoclinic unit cell is defined by a = 5.03 Å, b = 7.40 Å, c = 160.77 Å, β = 91.18 º. The *P2/c* orthorhombic unit cell is defined by a = 4.96 Å, b = 7.42 Å, c = 82.59 Å.

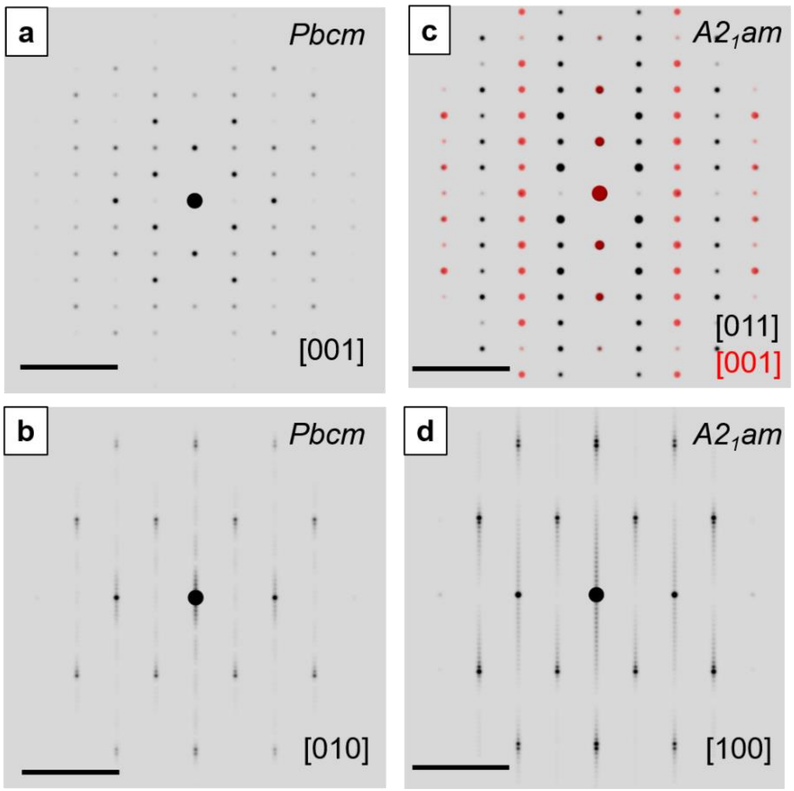

Figure S5: Simulated diffraction patterns for $C_{31}H_{64}$ with *Pbcm* symmetry (a,b), $C_{31}H_{64}$ with *$A2_1am$* symmetry (c,d). Diffraction patterns corresponding to the 'down-chain' orientated chains are found in (a,c) and diffraction patterns corresponding to the 'chains-flat' orientated chains are found in (b,d). The diffraction patterns were simulated using SingleCrystal, part of the CrystalMaker software. The scale bars indicate 0.5 Å^{-1}.

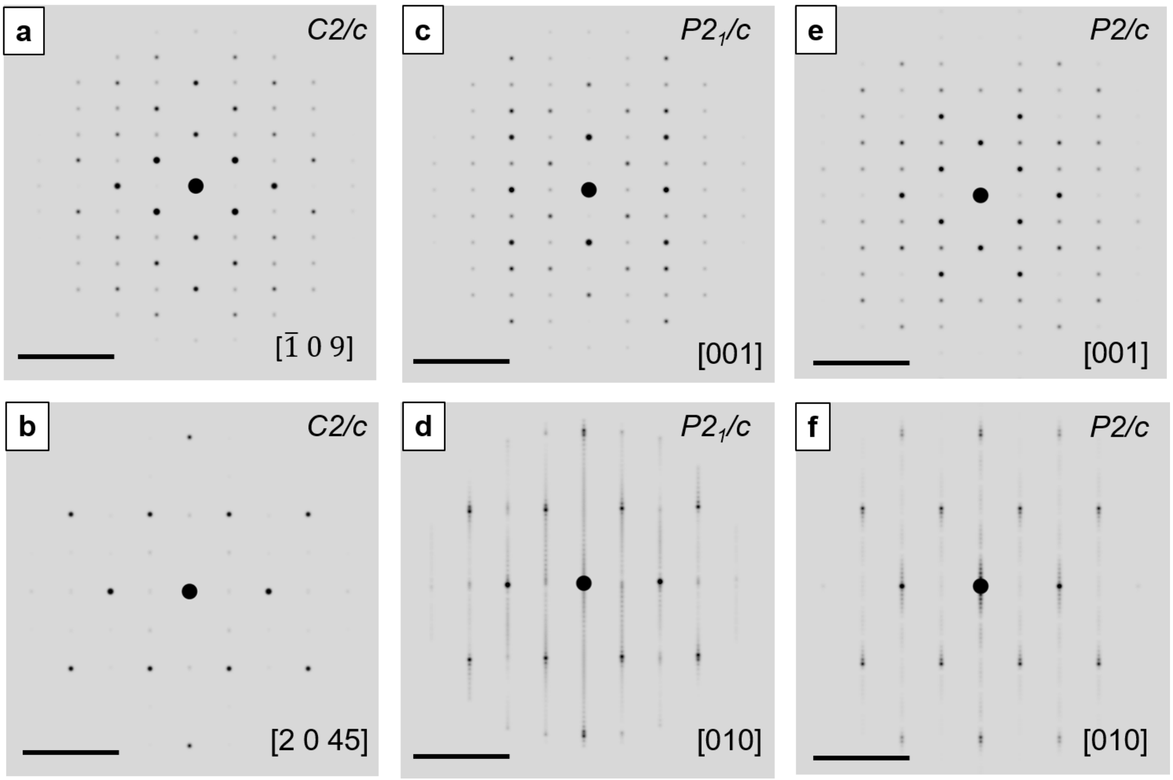

Figure S6: Simulated diffraction patterns for $C_{30}H_{61}OH$ with *C2/c* symmetry (a,b), *P2₁c* symmetry (c,d) and *P2/c* symmetry. Diffraction patterns corresponding to the 'down-chain' orientated chains are found in (a,c,e) and diffraction patterns corresponding to the 'chains-flat' orientated chains are found in (b,d,f). The diffraction patterns were simulated using SingleCrystal, part of the CrystalMaker software. The scale bars indicate 0.5 Å^{-1}.

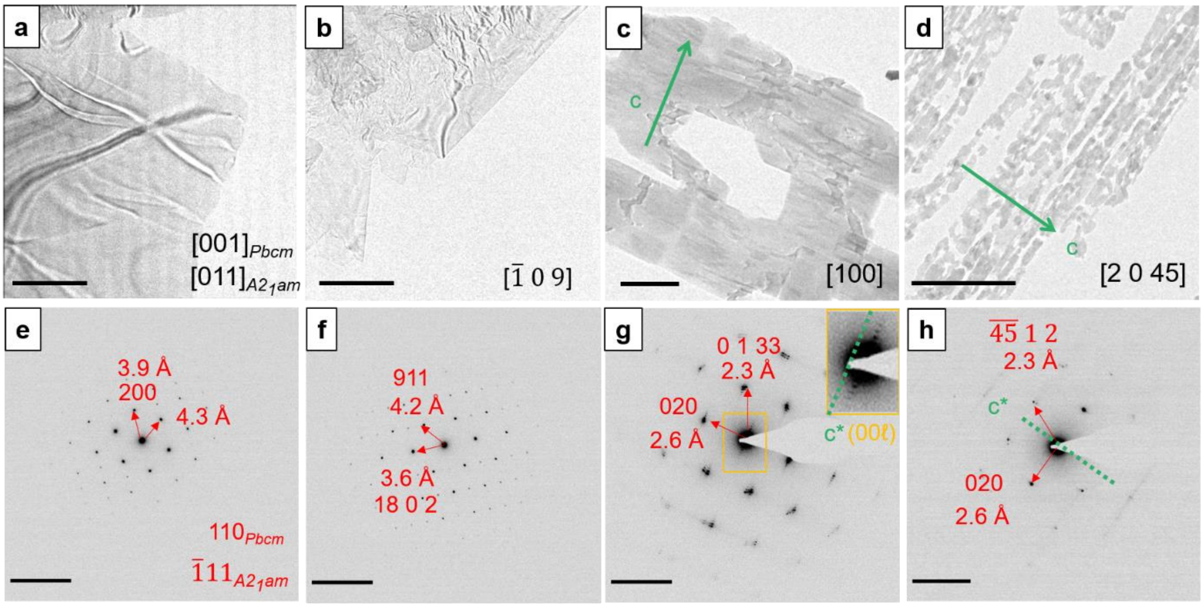

Figure S7: (a)-(d) Bright field (BF) TEM images and (e)-(h) selected area electron diffraction (SAED) patterns for (a,e) $C_{31}H_{64}$ prepared to align the alkyl chains parallel to the electron beam ('down-chain' orientation), (b,f) $C_{30}H_{61}OH$ prepared in the 'down-chain' orientation, (c,g) $C_{31}H_{64}$ prepared to align alkyl chains perpendicular to the electron beam ('chains-flat' orientation), and (d,h) $C_{30}H_{61}OH$ prepared in the 'chains-flat' orientation. Green arrows indicate the *c*-axis direction (alkyl chain axis) following the polyethylene cell (c_{PE}). Arrows overlaid on the SAED patterns mark the indexation of the patterns to the labelled respecitve indexed orientation. The inset in (g) shows an expanded section of the SAED pattern marked by the yellow box in (g), with the c^*_{PE} reciprocal lattice direction marked by the green dashed line. The BF image scale bars indicate 2 µm. The SAED pattern scale bars indicate 0.5 Å^{-1}.

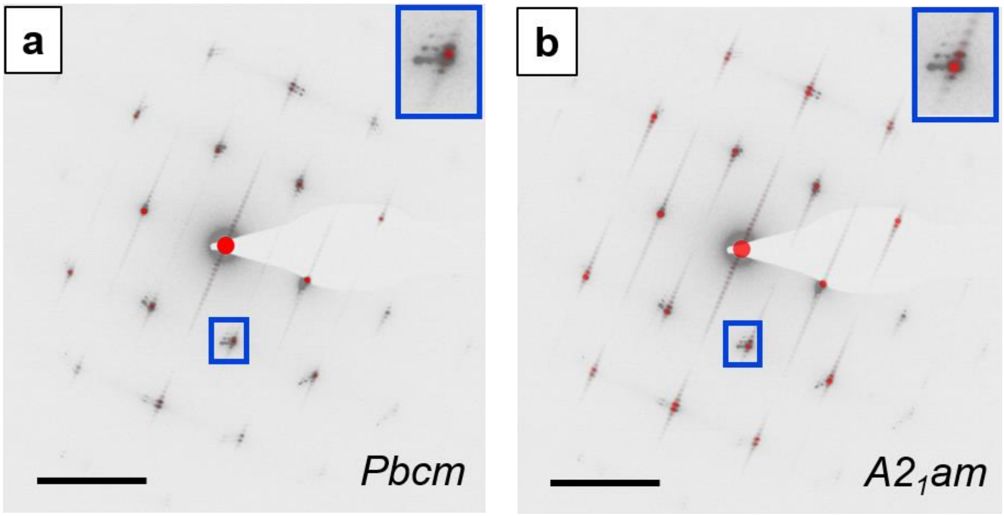

Figure S8: Simulated diffraction patterns (red) overlaid on experimental data for the 'chains-flat' orientated $C_{31}H_{64}$ chains. Insets show the magnified 01*l* spots. The diffraction patterns were simulated using SingleCrystal, part of the CrystalMaker software. The scale bars indicate 0.5 Å^{-1}.

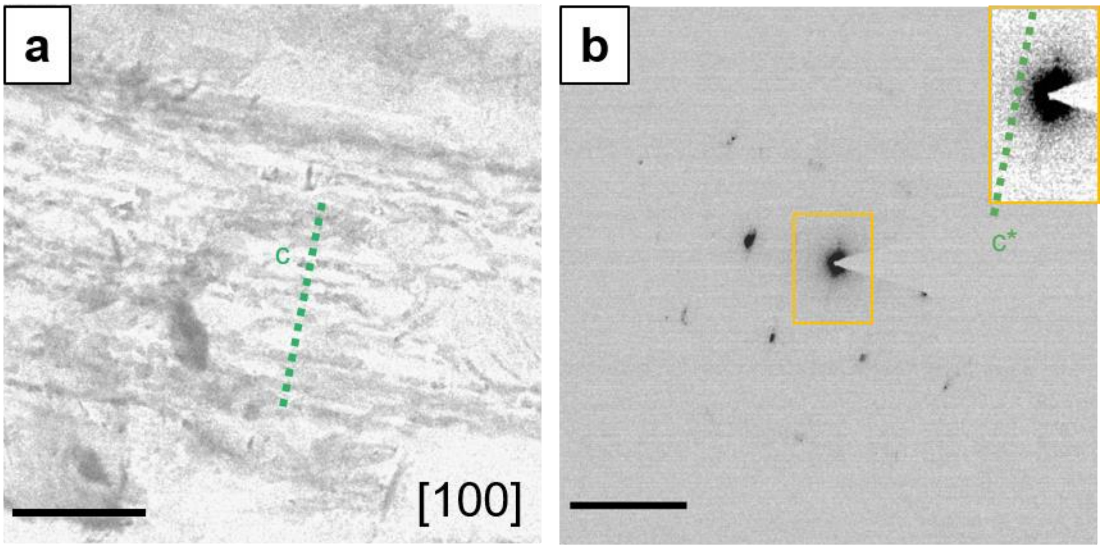

Figure S9: A BF image of $C_{30}H_{61}OH$ flat chain epitaxially prepared crystals and the corresponding SAED pattern showing an example of 00*l* spots. The BF image scale bar indicates 2 μm and the diffraction pattern scale bar indicates 0.5 Å^{-1}.

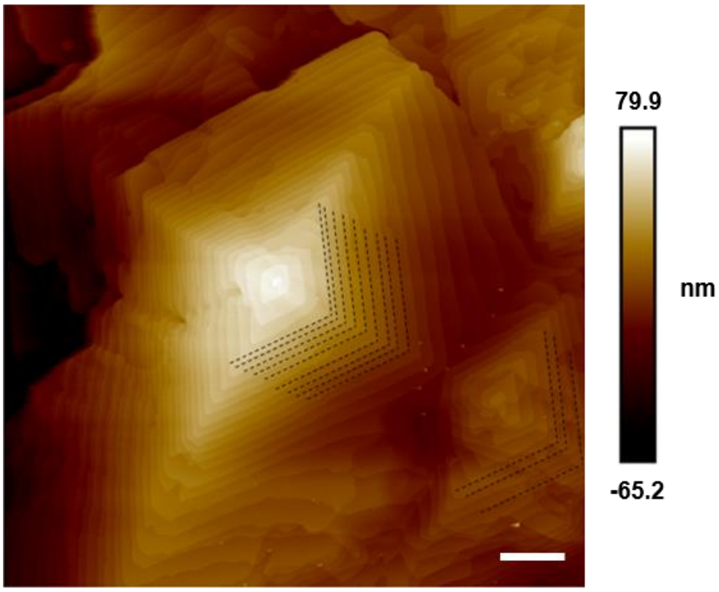

Figure S10: AFM height map of a $C_{31}H_{64}$ crystal. Scale bar indicates 4 µm. Dashed lines mark angle measurements of a series of terraces, with an estimated angle of 109°.

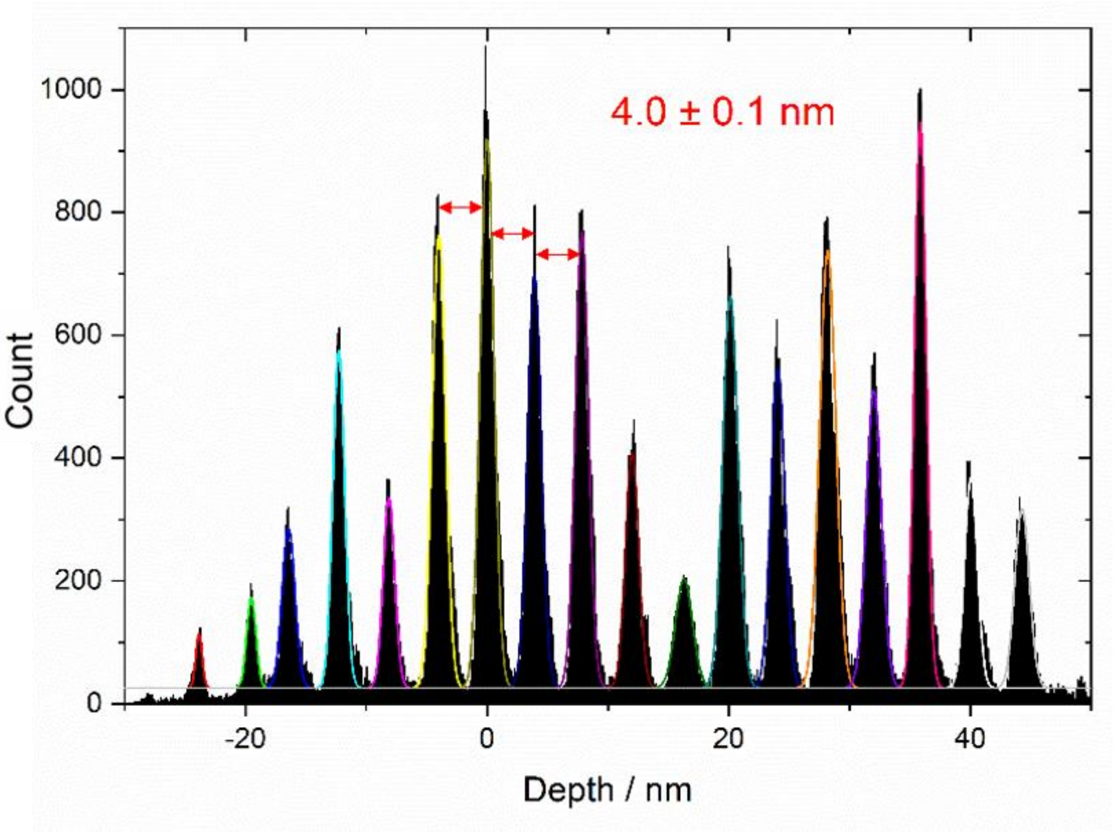

Figure S11: Histogram showing the height distribution across the $C_{31}H_{64}$ sample as measured by AFM. Height information for every pixel was extracted from the image shown in Figure 5a, and plotted. The histogram was fitted with a multi-peak Gaussian fit and the difference between peaks, indicated by the red arrows, and therefore the height difference between all 18 peaks, was calculated to be 4.0 ± 0.1 nm.

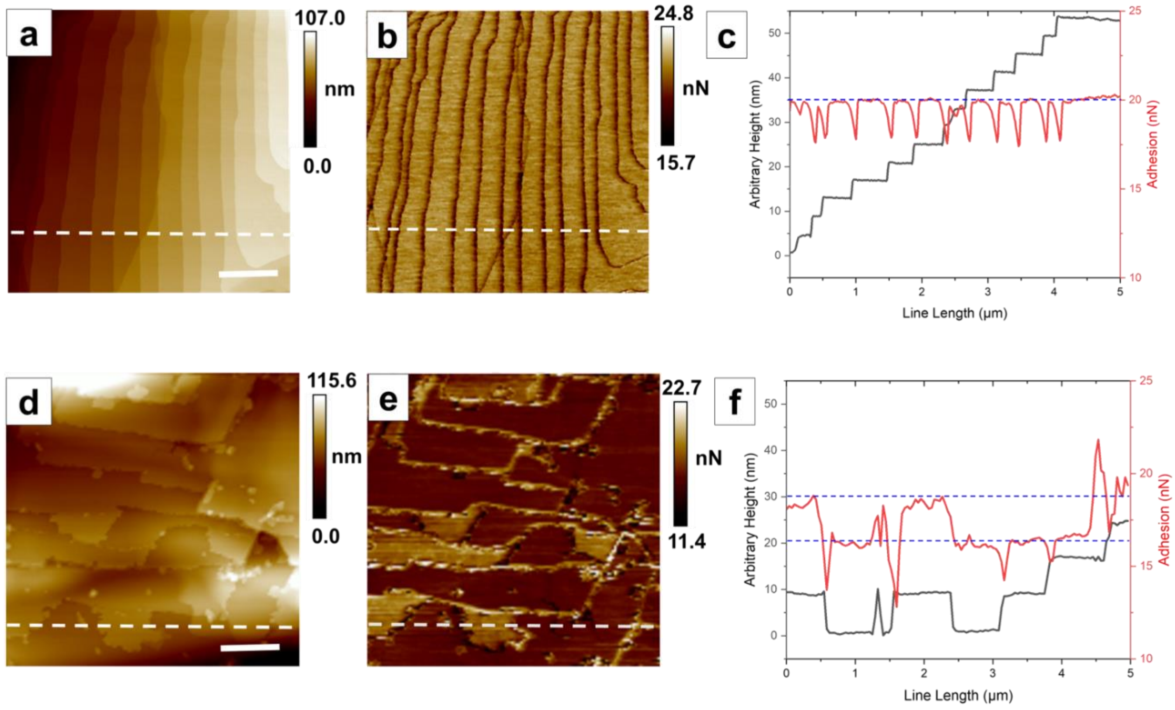

Figure S12: Height and adhesion profiles, with values along the indicated line plotted, obtained through AFM for $C_{31}H_{64}$ (a-c) and $C_{30}H_{61}OH$ (d-f) samples. Blue dotted lines indicate the variation in adhesion values across both samples. The scale bars indicate 2 μm.

Table S1: Critical dose or fluence of 300 keV electrons (C_F) (e$^-$/Å^2) that produces significant damage to the structure of these synthetic waxes as monitored by SAED Bragg diffraction spot decay, SED Bragg diffraction spot decay and as monitored by integrated intensity regions of interest from high angle ADF-STEM images. Uncertainties are given as the standard error estimated from replicate measurements across several reflections within a diffraction pattern and across multiple crystals.

Sample	C_F SAED	C_F SED	C_F vADF
n-hentriacontane ($C_{31}H_{64}$)	5.5 (± 0.7)	15.1 (± 0.7)	250 (± 80)
1-triacontanol ($C_{30}H_{61}OH$)	6 (± 2)	10.9 (± 0.9)	230 (± 30)

Table S2: Calculated standard deviation and standard error for each method and sample. Measurements are taken from individual spots within the same diffraction pattern and across multiple crystals. The measurements are grouped by the d-spacings of the scattering vectors corresponding to the planes {hkl}. *N* is the number of measurements and *d* is the d-spacing of the set of lattice planes.

<table>
<tr><th>Method</th><th>Sample</th><th>d (Å)</th><th>{hkl}</th><th>Critical fluence average ($e^-/Å^2$)</th><th>Standard Deviation ($e^-/Å^2$)</th><th>N</th><th>Standard Error ($e^-/Å^2$)</th></tr>
<tr><td rowspan="7">SAED</td><td rowspan="4">n-hentriacontane ($C_{31}H_{64}$)</td><td>4.3</td><td>{110}</td><td>7.1</td><td>3.2</td><td>28</td><td>0.6</td></tr>
<tr><td>3.9</td><td>{200}</td><td>5.4</td><td>3.1</td><td>16</td><td>0.8</td></tr>
<tr><td>2.2</td><td>{020}</td><td>5.5</td><td>1.6</td><td>10</td><td>0.5</td></tr>
<tr><td>1.9</td><td>{400}</td><td>4.1</td><td>1.2</td><td>2</td><td>0.8</td></tr>
<tr><td rowspan="3">1-triacontanol ($C_{30}H_{61}OH$)</td><td>4.3</td><td>{110}</td><td>8.3</td><td>1.8</td><td>16</td><td>0.5</td></tr>
<tr><td>3.9</td><td>{200}</td><td>6.4</td><td>1.4</td><td>8</td><td>0.5</td></tr>
<tr><td>2.2</td><td>{020}</td><td>4.5</td><td>4.9</td><td>2</td><td>3.5</td></tr>
<tr><td rowspan="6">SED</td><td rowspan="3">n-hentriacontane ($C_{31}H_{64}$)</td><td>4.3</td><td>{110}</td><td>14.5</td><td>1.8</td><td>4</td><td>0.9</td></tr>
<tr><td>3.9</td><td>{200}</td><td>14.4</td><td>0.6</td><td>2</td><td>0.5</td></tr>
<tr><td>2.2</td><td>{020}</td><td>16.4</td><td>1.0</td><td>2</td><td>0.7</td></tr>
<tr><td rowspan="3">1-triacontanol ($C_{30}H_{61}OH$)</td><td>4.3</td><td>{110}</td><td>10.0</td><td>0.7</td><td>8</td><td>0.2</td></tr>
<tr><td>3.9</td><td>{200}</td><td>11.0</td><td>1.5</td><td>4</td><td>0.7</td></tr>
<tr><td>2.2</td><td>{020}</td><td>11.7</td><td>3.8</td><td>4</td><td>1.9</td></tr>
<tr><td rowspan="2">vADF</td><td>n-hentriacontane ($C_{31}H_{64}$)</td><td>-</td><td>-</td><td>247</td><td>110</td><td>2</td><td>80</td></tr>
<tr><td>1-triacontanol ($C_{30}H_{61}OH$)</td><td>-</td><td>-</td><td>226</td><td>43</td><td>2</td><td>31</td></tr>
</table>

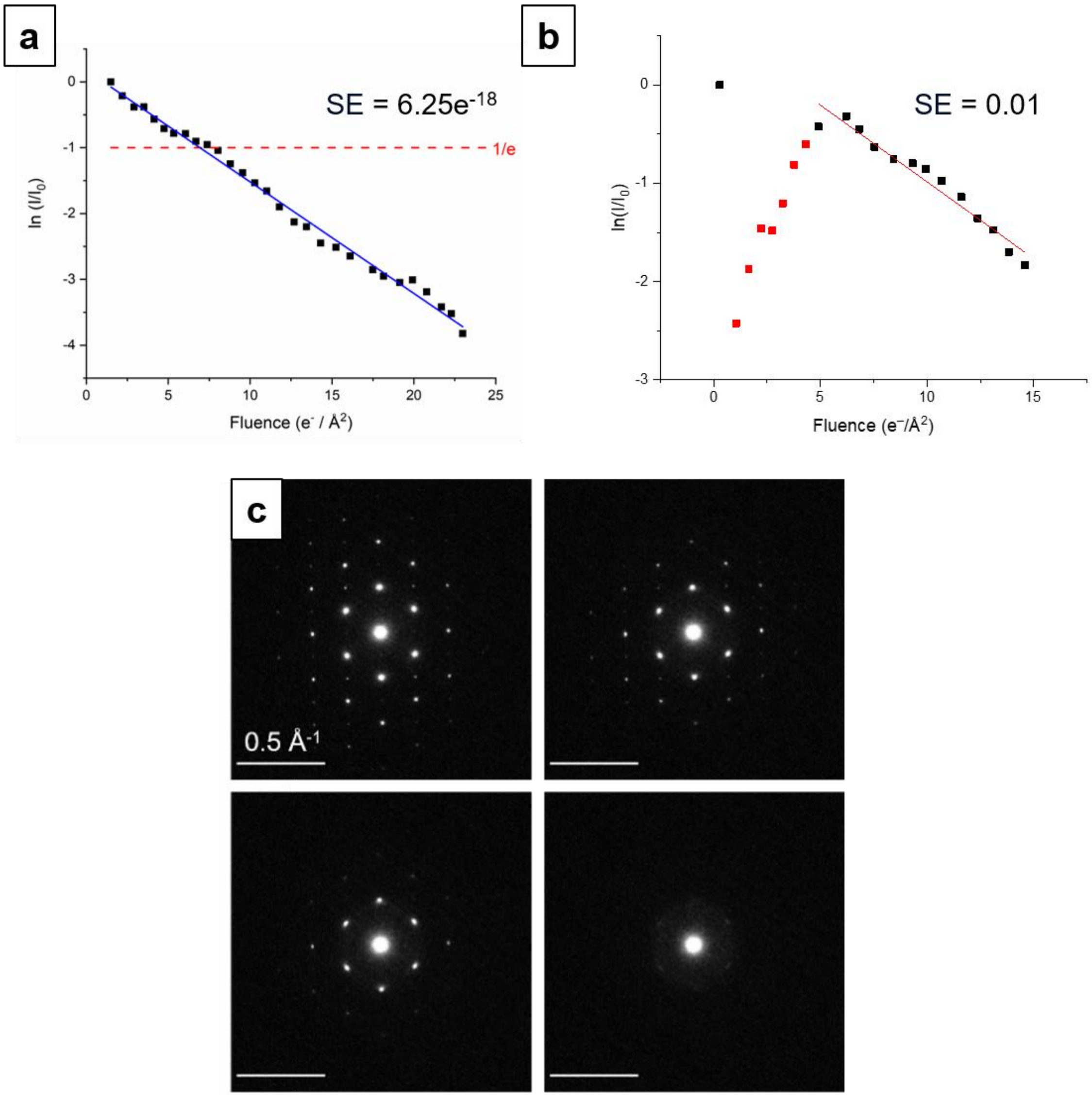

Figure S13: (a, b) Plots of the natural logarithm of the decay of the normalised intensity of a diffraction spot versus total accumulated fluence for two samples. The standard error (SE) of the slope term in the fit is shown in each case. The plot in (a) exhibits an exponential decay from the first measurement of the intensity. The plot in (b) exhibits an initial rise before a subsequent exponential decay (linear region in the logarithm linearised data). Such responses reflect initial reorientation under the beam.[1] The critical fluence C_F is calculated from gradient of linear fit (blue). $I = I_0 \exp(-\tau D)$ for intensity *I* and fluence or dose *D* and decay rate τ at $I/I_0 = 1/e$. (c) Diffraction patterns across a dose-damage time series illustrating the decay of Bragg spots. Scale bars in (c) indicate 0.5 Å^{-1}.

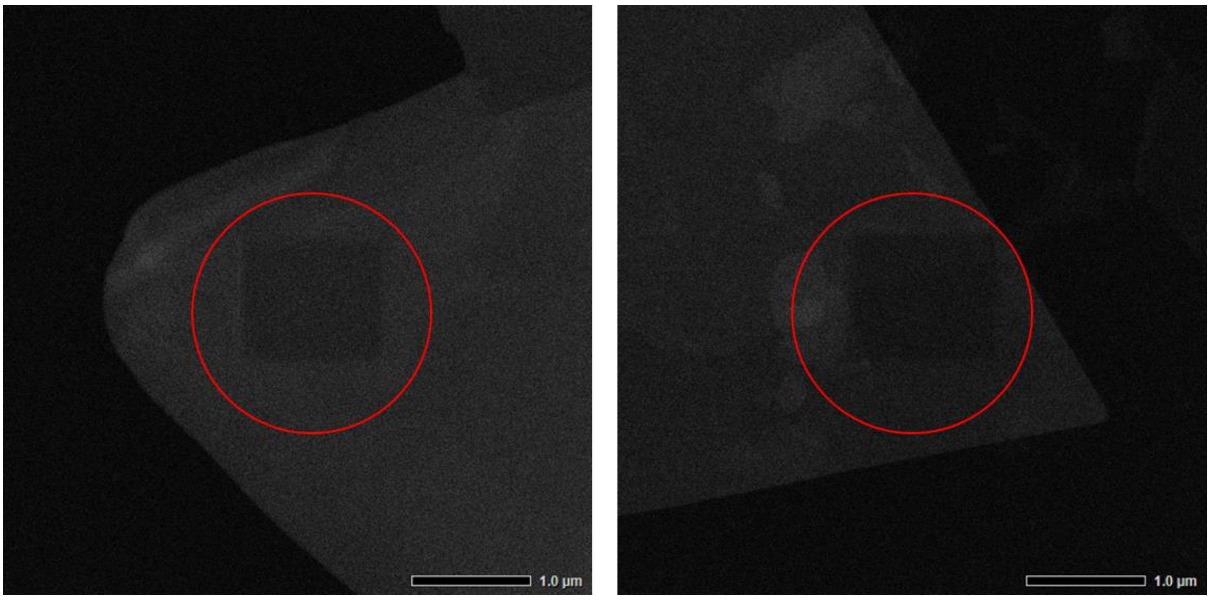

Figure S14: Images showing darker rectangular areas (circled in red) where the beam had probed in earlier measurements.

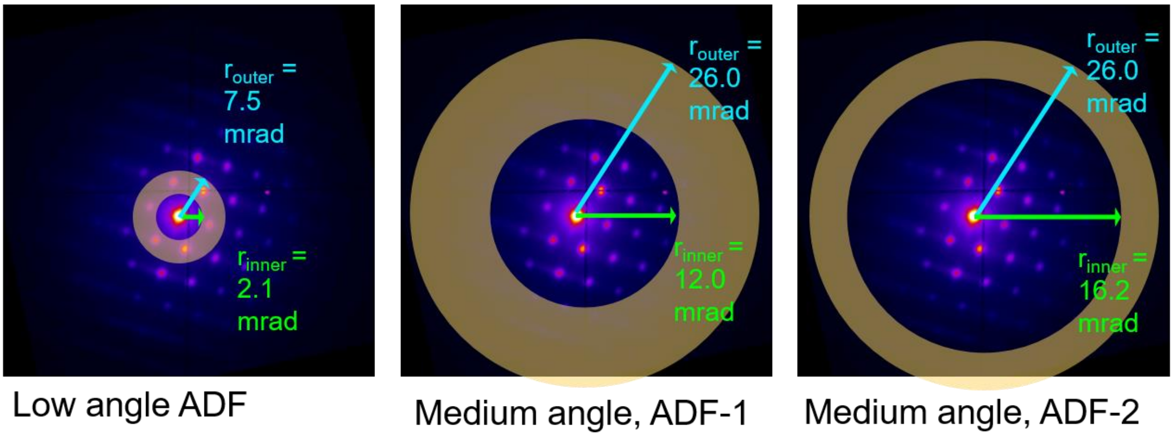

Figure S15: Virtual annular dark-field (VADF) imaging: a defined annular dark-field detector is placed on the diffraction pattern to integrate all intensities within the detector region. The figure presents the different detector regions used.

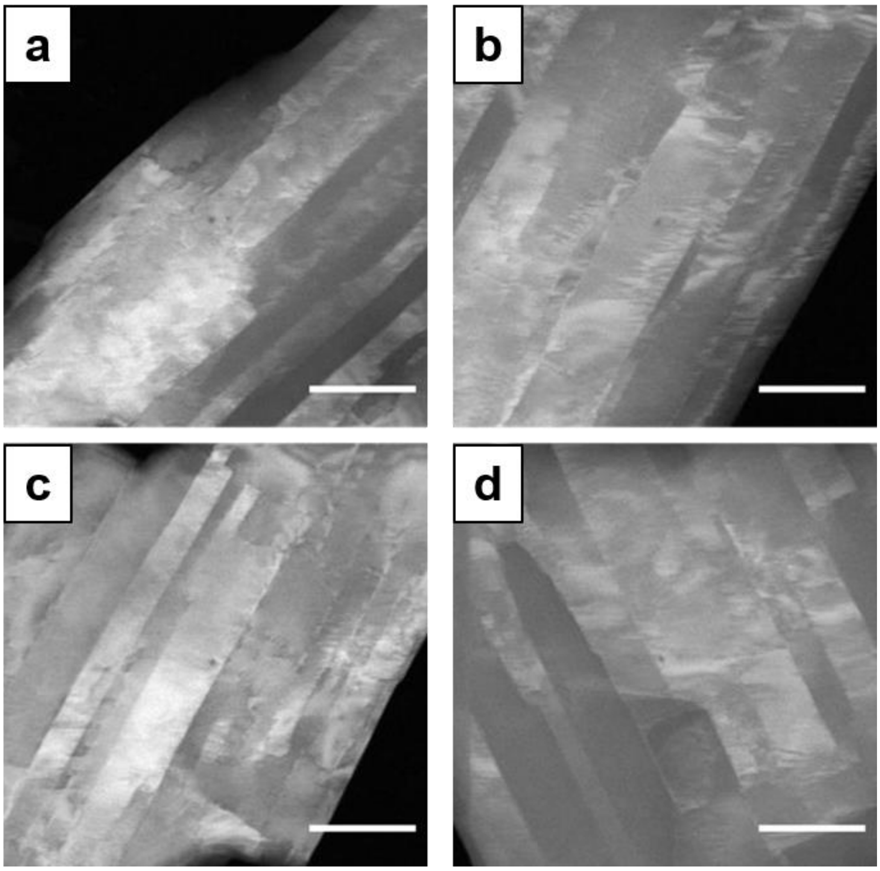

Figure S16: Low angle ADF images formed from four $C_{31}H_{64}$ epitaxially orientated crystals showing distinct grain structure. All scale bars indicate 500 nm.

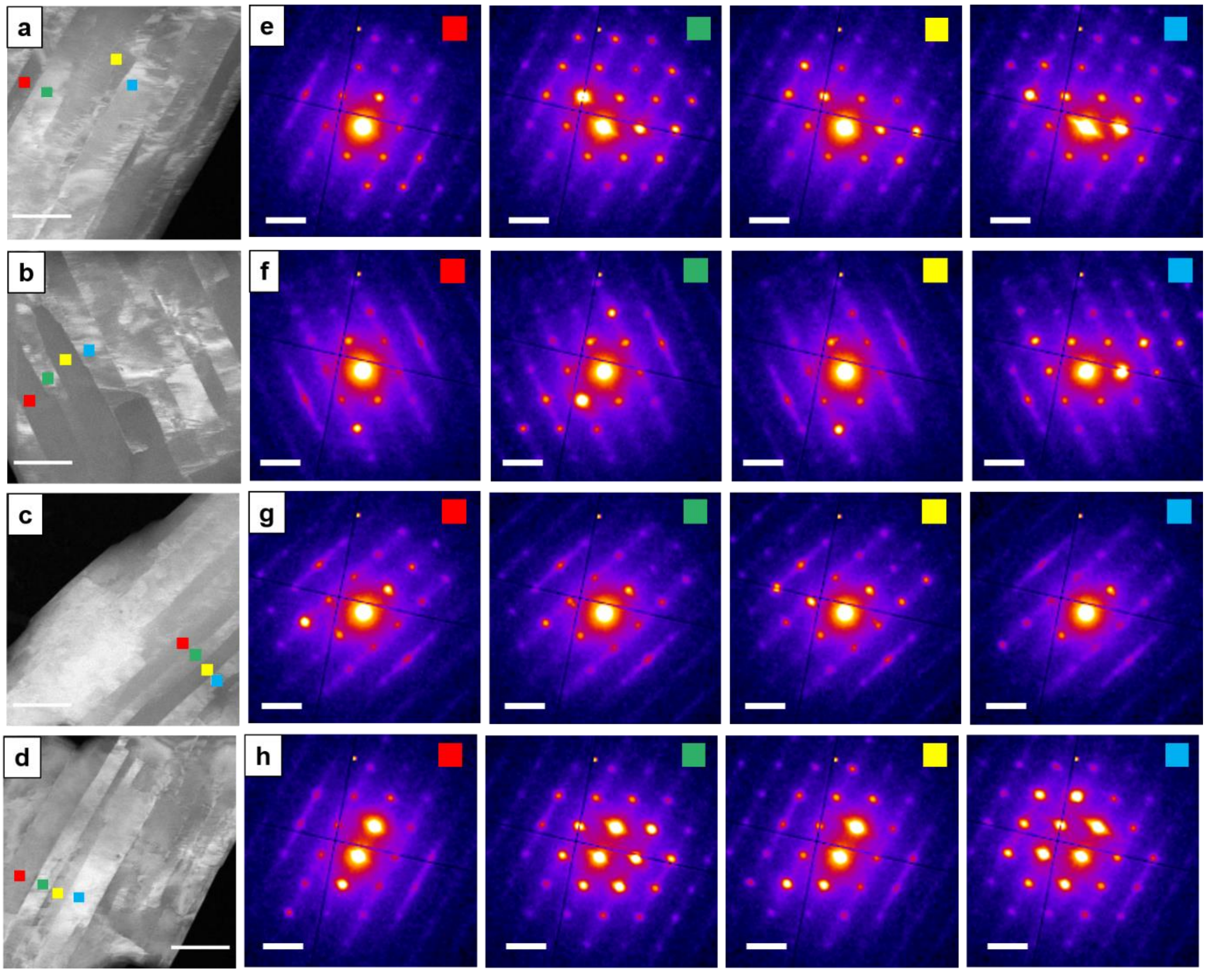

Figure S17: (a,e) (b,f) (c,g) (d,h) Summed diffraction patterns (e-h) from an area of 10 x 10 pixels shown by the coloured boxes in the corresponding [100] orientated n-hentriacontane ($C_{31}H_{64}$) crystals (a-d). All STEM image scale bars indicate 500 nm. All diffraction pattern scale bars indicate 0.5 Å^{-1}.

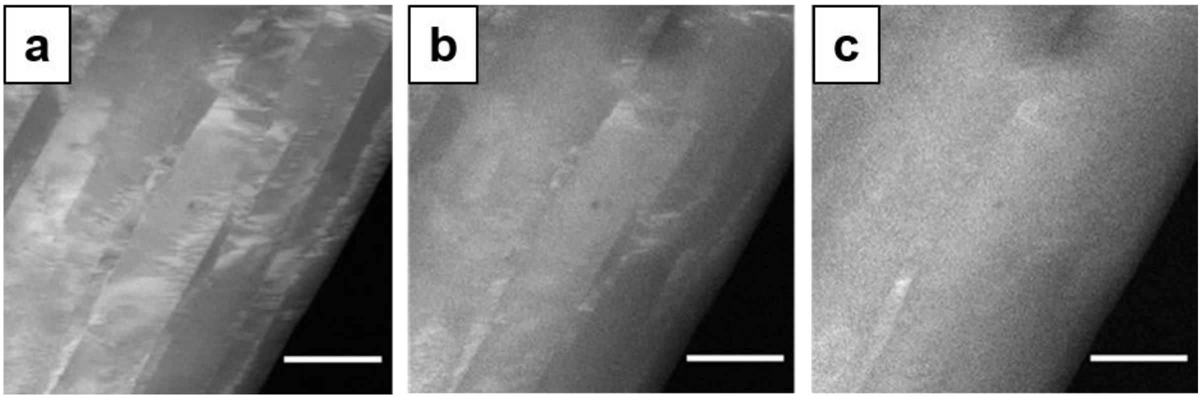

Figure S18: Images of a $C_{31}H_{64}$ epitaxially orientated crystal with increasing vADF angle, (a) low angle ADF (b) medium angle, ADF-1 (c) medium angle, ADF-2. All scale bars indicate 500 nm.

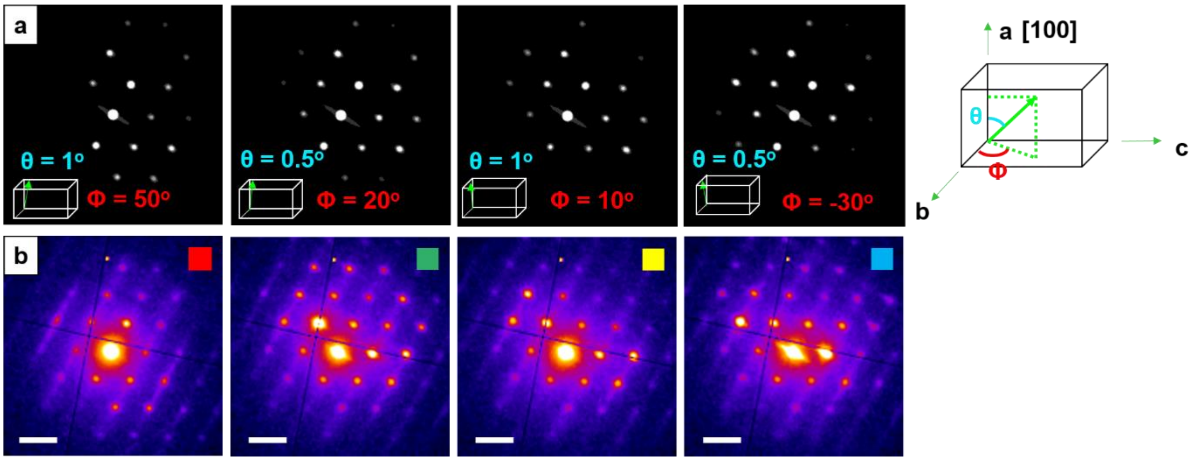

Figure S19: (a) Diffraction patterns simulated using Pyxem to apply a defined tilt to the diffraction pattern produced by the $C_{31}H_{64}$ crystals. These are compared to diffraction patterns extracted from smaller areas within a $C_{31}H_{64}$ dataset seen in Figure 6. The schematic shows how theta and phi are defined relative to the crystal axis. The green arrows show a schematic representation of a relative tilt between the electron beam and the unit cell. The scale bars indicate 0.5 Å^{-1}.

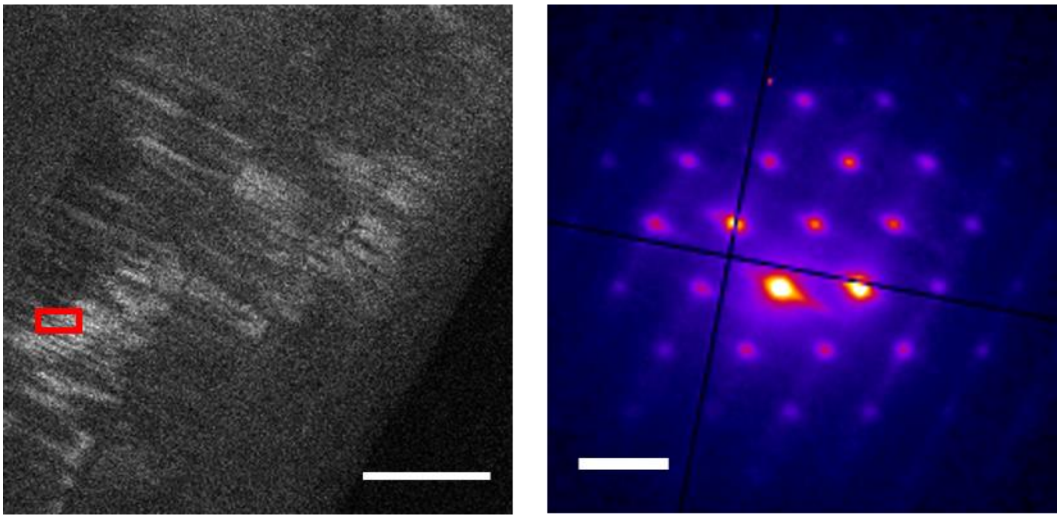

Figure S20: Diffraction pattern extracted from a region where the sharp intensity lines were seen in the vdf produced from the 00*l* signal areas, indicated by the red box. The 01*l* peaks have an elliptical shape suggesting splitting of these peaks, showing lamellar ordering is found within these regions. The image scale bar (left) indicates 500 nm. The diffraction pattern scale bar (right) indicates 0.5 Å^{-1}.

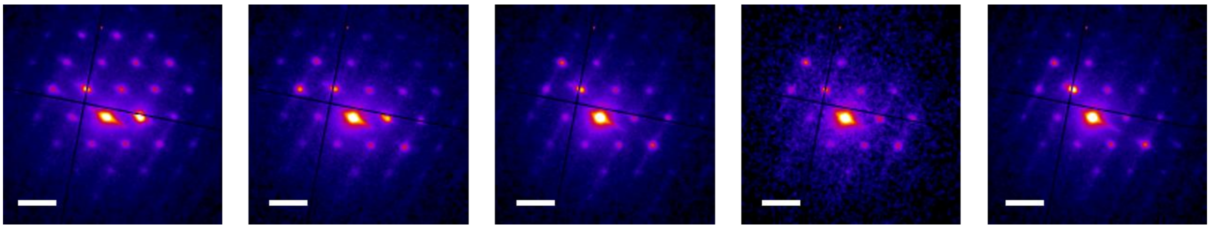

Figure S21: Diffraction patterns extracted from areas where the sharp intensity lines were seen in the vdf produced from the 00*l* signal areas in Figure S20. The scale bars indicate 0.5 Å^{-1}.

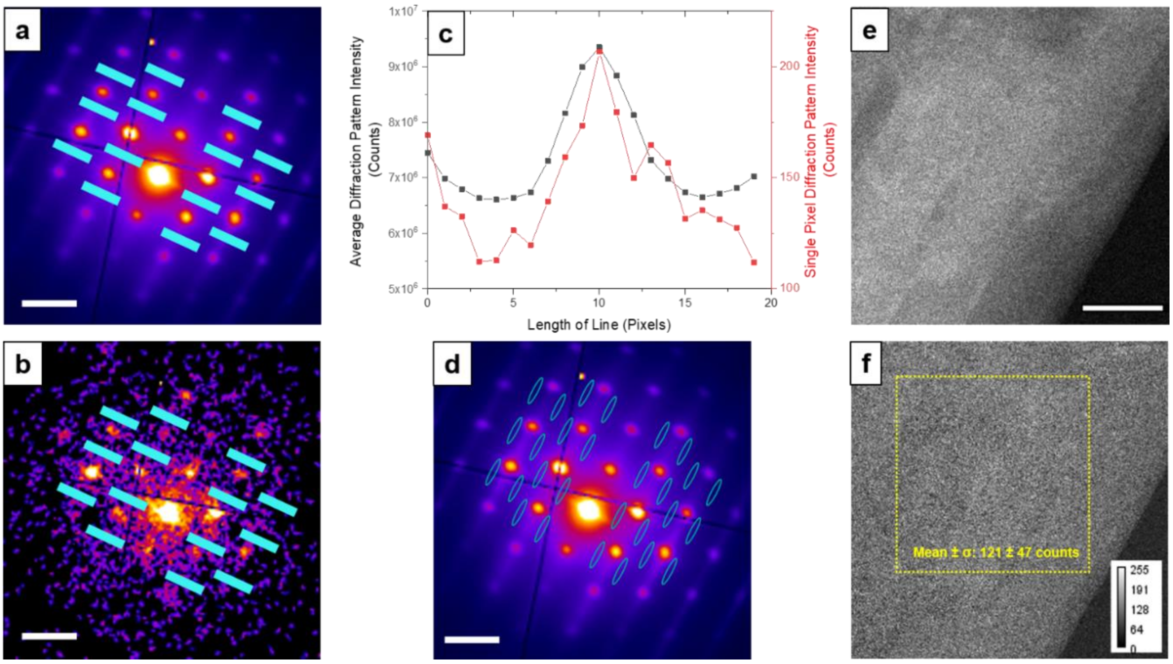

Figure S22: An analysis of the signal in the diffuse scattering signal encoded in the VDF image in Figure 7d. (a) Integrated diffraction pattern and (b) a single pixel diffraction pattern extracted from the datset shown in Figure 7. The light blue lines mark the positions of a series of line profiles used to extracted and sum the diffuse scattering signal and local background, depicted in the form of intensity line profiles in (c). The integration over these line profiles mimics the selection of matched virtual apertures in Figure 7. In (c) the black trace shows the integrated line profile for the integrated pattern and the red trace shows the integrated line profile for the single-pixel pattern. Evaluation of the signal-to-background features are shown in Table S3. (d) Ovals mark the positions of virtual apertures used to form a VDF estimating the background off the diffuse scattering streaks (taken as double the background counts for Figure 7d given twice the apertures). (f) A VDF image formed by subtracting the estimated background intensity from the VDF in Figure 7d (diffuse scattering signal counts). A dashed square marks an area used for inspection of the counts encoded in the grayscale image. The average of the background-subtracted counts in the yellow square is ~120. The image in the main text Figure 7d has an average of ~ 260 counts, and the image in (e) in this figure has an average of ~ 140, indicating (f) in this figure is statistically separable from the background. Scale bars in (a), (b), and (d) indicate 0.5 Å^{-1}. The scale bar in (e) indicates 500 nm.

Table S3: Signal-to-background evaluation in diffuse scattering signals. The integrated SED refers to the black curve in Figure S22. The single-pixel SED refers to the red curve in Figure S22. The estimated background counts (counts on Merlin-Medipix detector) are given as orders of magnitude for the integrated pattern for simplicity of presentation of large values. Counts are recorded as integer-precision values. For Poisson statistics, the standard deviation σ = √N for N counts. Probabilities were calculated using the Scipy poisson.sf() survival function.

**Measurement**	**$N_{background}$ (counts)**	**3σ (3√N)**	**N_{peak} (counts)**	**Probability of observing peak counts for only noisy background recorded (1-sided tail, Poisson distribution)**
Integrated SED	7×10^6	8×10^3	9×10^6	0
Single pixel SED	112	32	207	$3.5e^{-16}$

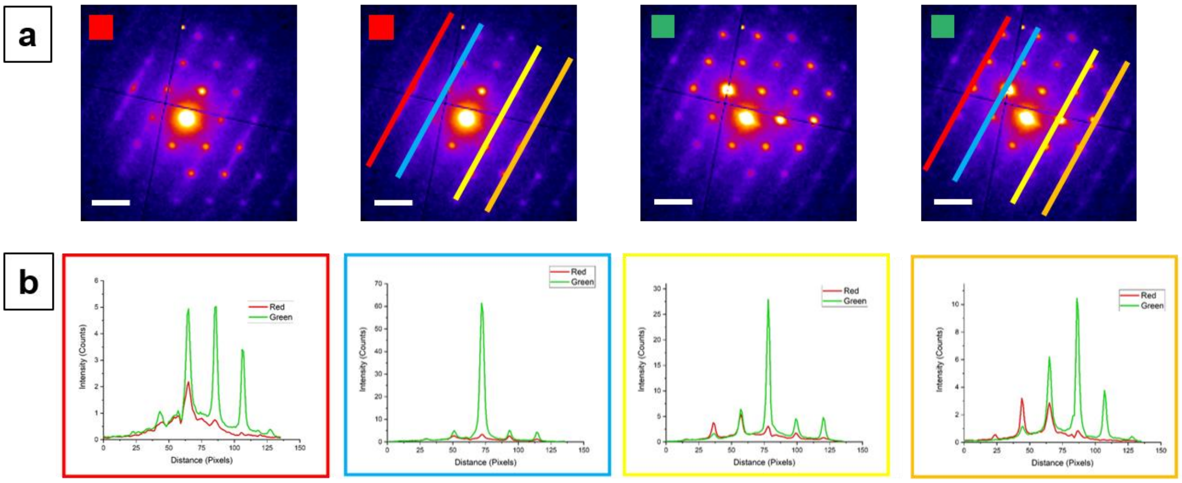

Figure S23: Line profiles across (hkl) reflections measuring intensity corresponding to the areas outlined by the red and green boxes in Figure 6. The diffraction pattern scale bars indicate 0.5 Å^{-1}.

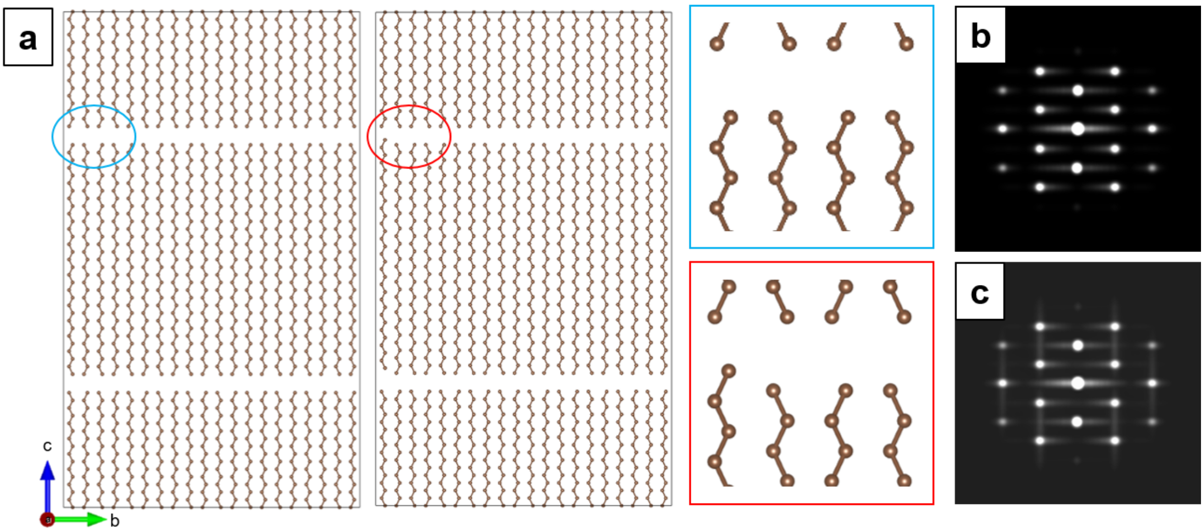

Figure S24: (a) A supercell structure constructed by extending the $C_{31}H_{64}$ *$A2_1am$* unit cell along the *b*-axis to give a total of 10 repeats (the symmetry was reduced to *P1* in all supercell models). The left-hand structure depicts a 'perfect' supercell, and the right-hand structure depicts a supercell with a single molecule displaced along the *c*-axis (along the chain axis). The blue and red circled regions have been enlarged to clearly show the displaced chain. (b) A simulated diffraction pattern of the 'perfect' supercell, matching simulations for a single $C_{31}H_{64}$ *$A2_1am$* unit cell. (c) A calculated diffraction pattern constructed by overlaying three kinematical from supercell structures with 10, 15 and 20 repeats along the *b*-axis, each with a single chain displaced along the *c*-axis to emulate a distribution of displacement periodicities. In this diffraction pattern, diffuse scattering (perpendicular to the 00*l* spots) can be seen only at 0*kl* for *l* ≠ 0, in alignment with previous models of sliding mode disorder and with experimental observations. Patterns in (b) and (c) were simulated using the Pyxem Python package.

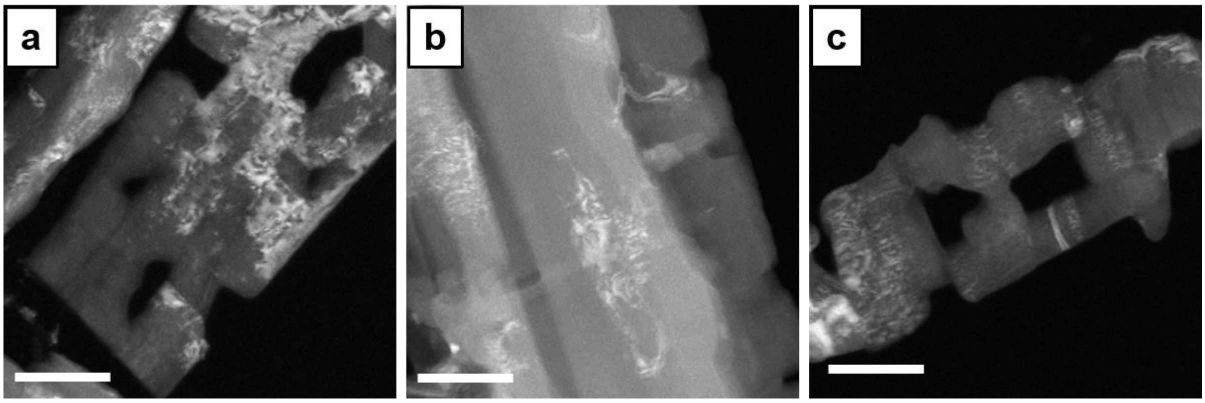

Figure S25: Low angle ADF images formed from four $C_{30}H_{61}OH$ epitaxially orientated crystals showing no distinct grain structure. All scale bars indicate 500 nm.

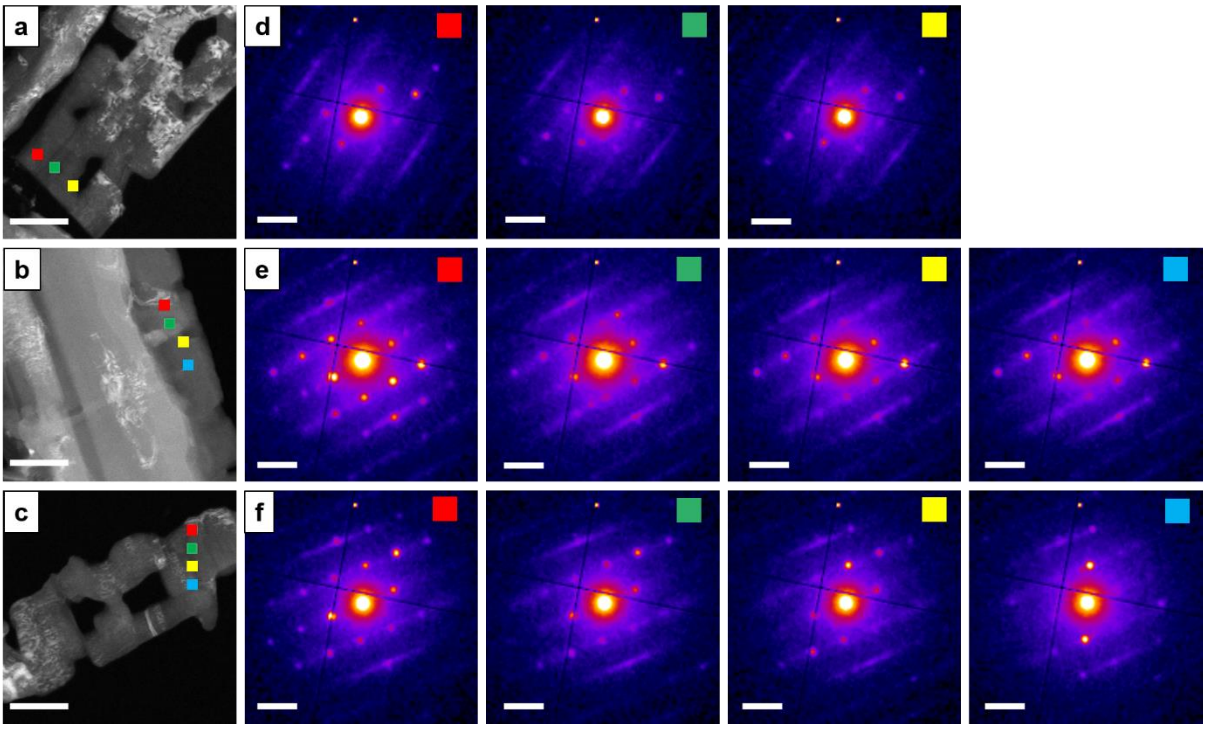

Figure S26: (a,d) (b,e) (c,f) Summed diffraction patterns (d-f) from an area of 10 x 10 pixels shown by the outlined boxes in the corresponding [100] orientated 1-triacontanol ($C_{30}H_{61}OH$) crystals (a-c). All STEM image scale bars indicate 500 nm. All diffraction pattern scale bars indicate 0.5 Å^{-1}.

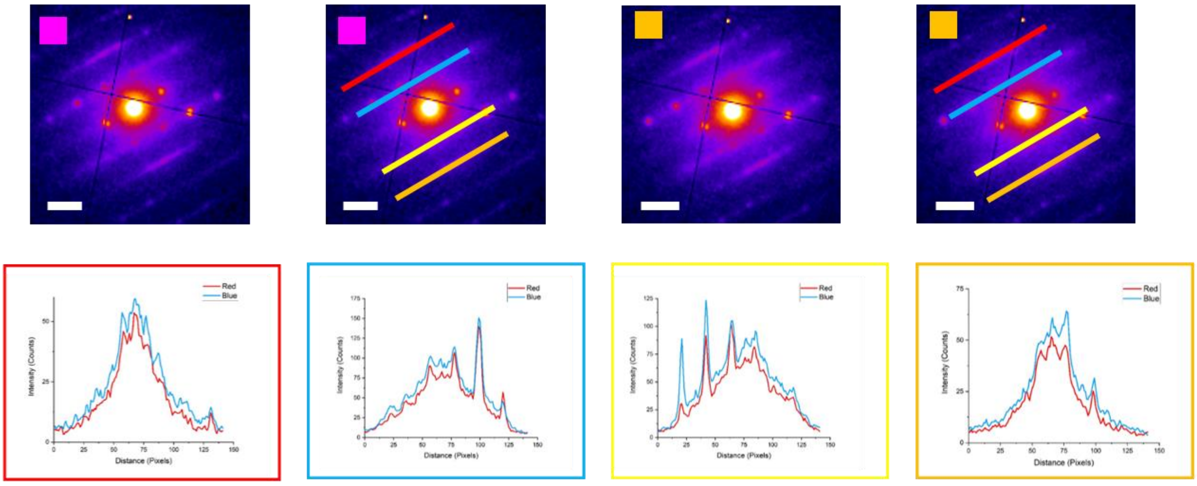

Figure S27: Line profiles across (hkl) reflections measuring intensity corresponding to the areas outlined by the pink and orange boxes in Figure S27. The diffraction pattern scale bars indicate 0.5 Å^{-1}.

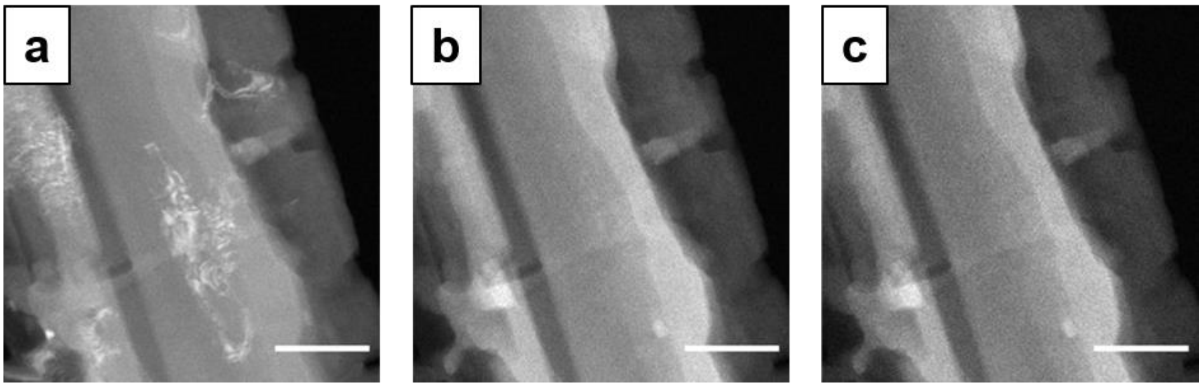

Figure S28: Images of a $C_{30}H_{61}OH$ epitaxially orientated crystal with increasing vADF angle, (a) low angle ADF (b) medium angle, ADF-1 (c) medium angle, ADF-2. All scale bars indicate 500 nm.

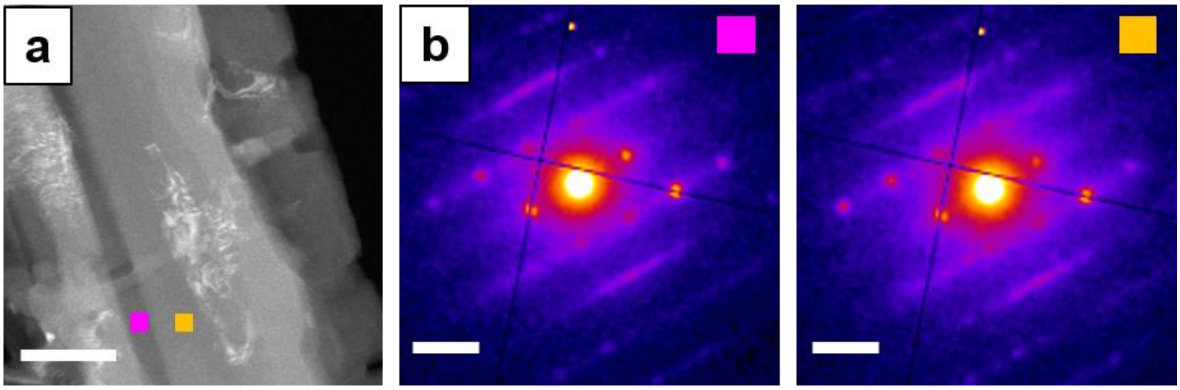

Figure S29: [100] orientated 1-triacontanol ($C_{30}H_{61}OH$) crystal(s). (b) Summed diffraction patterns from an area of 10 x 10 pixels shown by the outlined boxes in the [100] orientated 1-triacontanol ($C_{30}H_{61}OH$) crystal(s) (a). The ADF-STEM image scale bar in (a) indicates 500 nm. All diffraction pattern scale bars indicate 0.5 Å^{-1}.

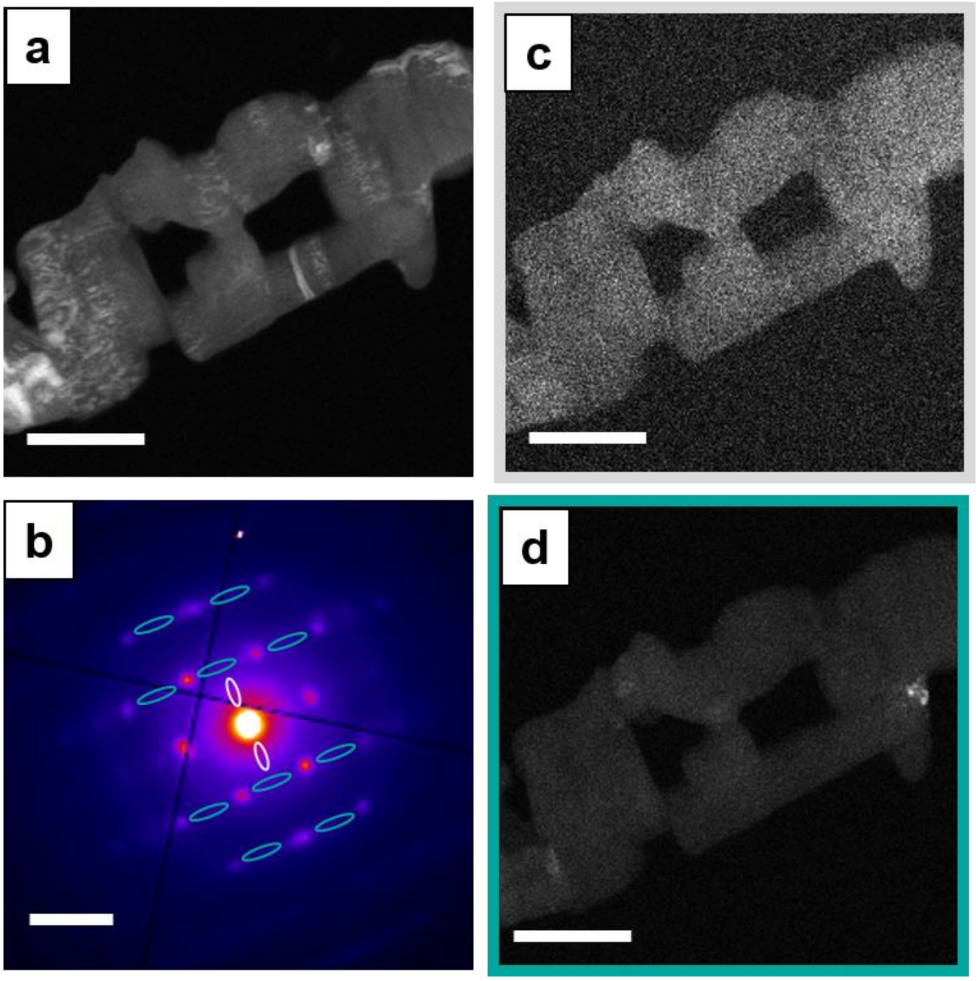

Figure S30: (a) Low angle ADF image of a $C_{30}H_{61}OH$ epitaxially orientated crystal. (b) Average diffraction pattern of crystal in (a). (c) Virtual dark field image produced from signal area defined by the grey ovals in the average diffraction pattern (b). (d) Virtual dark field image produced from diffuse scattering signal in the diffraction pattern defined by the blue ovals in the average diffraction pattern (b). The STEM image scale bars indicate 500 nm. The diffraction pattern scale bar in (b) indicates 0.5 Å^{-1}.

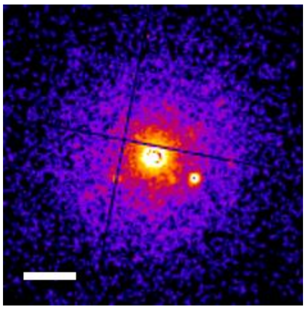

Figure S31: Summed diffraction pattern extracted from bright pixel areas in d in the $C_{30}H_{61}OH$ 00*l* vdf image. No distinct 00*l* lines can be seen around the central beam, although this pattern suffers from low signal to noise due to being summed over a low count of pixels. The scale bar indicates 0.5 Å^{-1}.

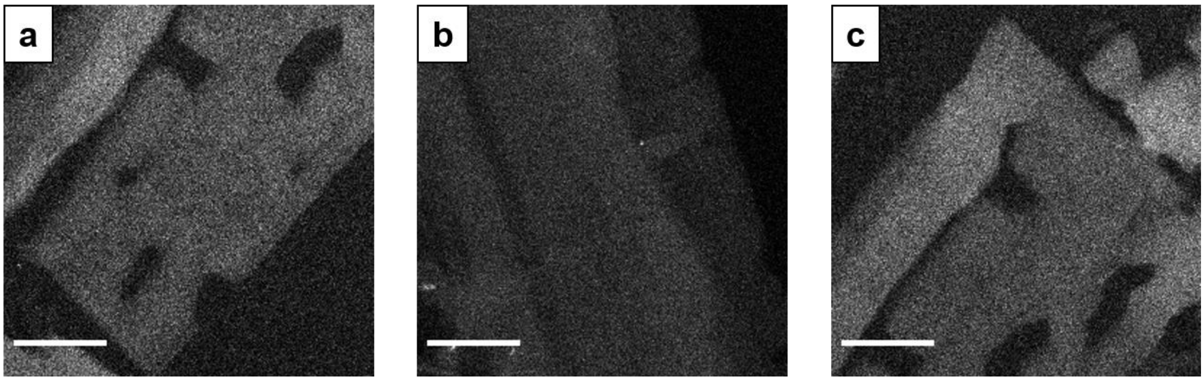

Figure S32: Virtual dark field images produced from signal area in the average diffraction pattern for each corresponding dataset along the c* direction next to the central beam (where a 00*l* line signal should arise). The scale bars indicate 500 nm.

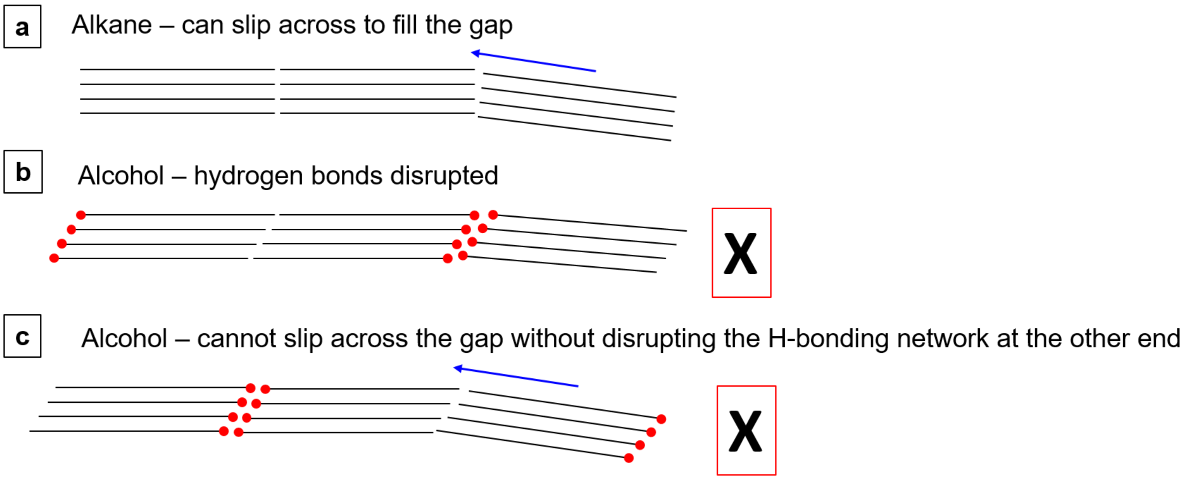

Figure S33: A simplified schematic to show a possible explanation why grain structure is lost in $C_{30}H_{61}OH$ vs $C_{31}H_{64}$. The black lines represent the carbon chains, with the red circle representing the alcohol group in $C_{30}H_{61}OH$. The $C_{31}H_{64}$ chains pack in straight lines end to end, whereas the $C_{30}H_{61}OH$ chains arrange with a "staircase" packing. With this staircase packing, there is a barrier to the chains tilting not seen in $C_{31}H_{64}$ (b and c). Notably, tilts between grains in $C_{31}H_{64}$ are approximately 0.5-1° (see Figure S19), consistent with energetically feasible structures like dislocation networks at low-angle grain boundaries or additional amorphous content at grain boundaries not resolved here.

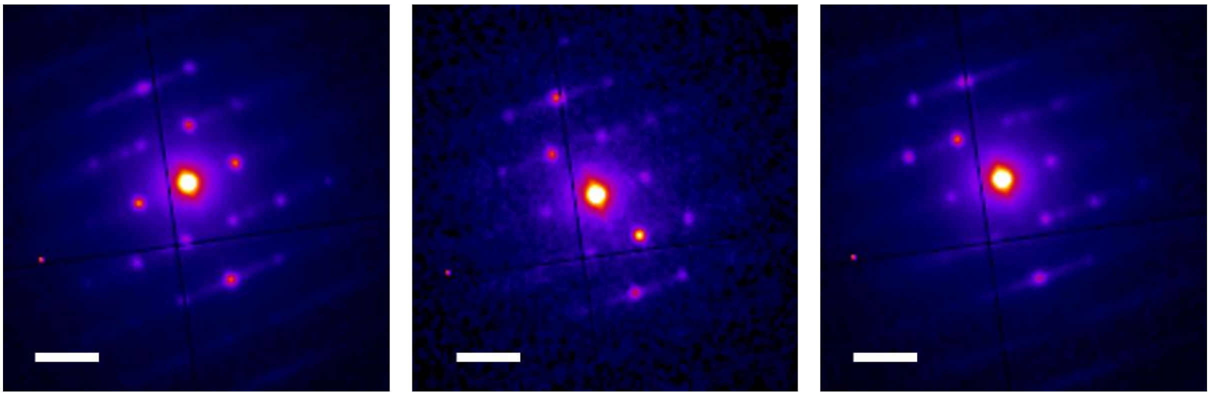

Figure S34: Summed diffraction pattern extracted from bright pixel areas in 15% $C_{30}H_{61}OH$ 00*l* vdf images (Figure 10). The scale bars indicate 0.5 Å^{-1}.

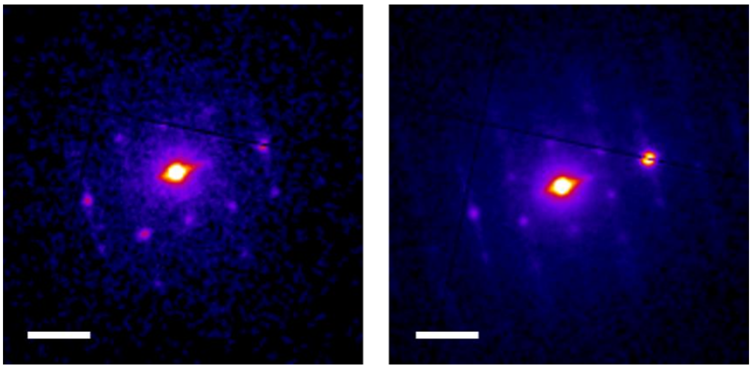

Figure S35: Summed diffraction pattern extracted from bright pixel areas in 30% $C_{30}H_{61}OH$ 00*l* vdf images (Figure 10). The scale bars indicate 0.5 Å^{-1}.

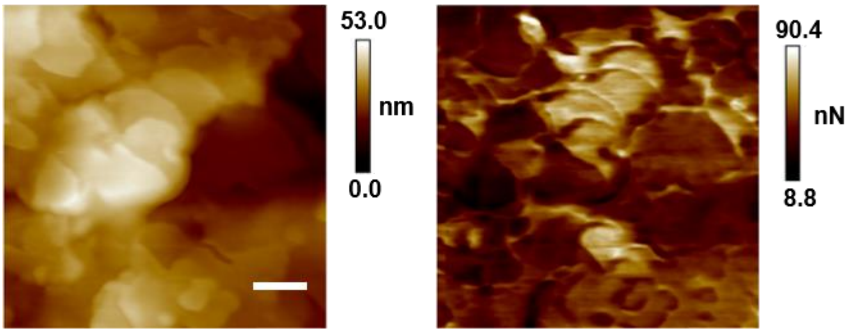

Figure S36: Height and adhesion profiles obtained through AFM. The scale bar indicates 250 nm. The higher magnification means bimodal adhesion variation is more clearly seen.

References

(1) S'Ari, M.; Blade, H.; Brydson, R.; Cosgrove, S. D.; Hondow, N.; Hughes, L. P.; Brown, A. Toward Developing a Predictive Approach to Assess Electron Beam Instability during Transmission Electron Microscopy of Drug Molecules. *Mol. Pharm.* **2018**, *15* (11), 5114–5123. https://doi.org/10.1021/acs.molpharmaceut.8b00693.
